# Piezo1 Impairs Endothelial Barrier and Drives Aortic Aneurysm and Dissection via STAT3-Dependent Activation of the CCL2–CCR2 Axis

**DOI:** 10.34133/research.1351

**Published:** 2026-07-09

**Authors:** Kehui Yang, Yang Liu, Xiaoran Huang, Dehui Hou, Han Du, Jingwen Wang, Hongwei Yue, Yunyun Guo, Sumei Cui, Huidan Zhang, Yijun Sun, Xin Li, Jun Ren, Feng Xu, Yuguo Chen

**Affiliations:** ^1^ Department of Emergency Medicine, Qilu Hospital, Shandong University, Jinan, China.; ^2^ Shandong Provincial Clinical Research Center for Emergency and Critical Care Medicine, Institute of Emergency and Critical Care Medicine of Shandong University, Chest Pain Center, Qilu Hospital of Shandong University, Jinan, China.; ^3^ Medical and Pharmaceutical Basic Research Innovation Center of Emergency and Critical Care Medicine, China’s Ministry of Education, Shandong Provincial Engineering Laboratory for Emergency and Critical Care Medicine, Key Laboratory of Emergency and Critical Care Medicine of Shandong Province, Key Laboratory of Cardiopulmonary-Cerebral Resuscitation Research of Shandong Province, Qilu Hospital of Shandong University, Jinan, China.; ^4^ NMPA Key Laboratory for Clinical Research and Evaluation of Innovative Drug, Qilu Hospital of Shandong University, Jinan, China.; ^5^ State Key Laboratory for Innovation and Transformation of Luobing Theory; The Key Laboratory of Cardiovascular Remodeling and Function Research, Chinese Ministry of Education, Chinese National Health Commission and Chinese Academy of Medical Sciences, Qilu Hospital of Shandong University, Jinan, China.; ^6^ Department of Emergency Medicine, Guangdong Provincial People’s Hospital, Guangdong Academy of Medical Sciences, Southern Medical University, Guangzhou, China.; ^7^ Department of Cardiology, Shanghai Institute of Cardiovascular Diseases, Zhongshan Hospital, Fudan University, Shanghai, China.

## Abstract

Endothelial cells (ECs) sense hemodynamic forces and critically influence aortic aneurysm and dissection (AAD) development. However, the possible contribution of the mechanical sensor Piezo1 to AAD remains elusive. Single-cell mRNA sequencing identified Piezo1 as the most up-regulated mechanical sensor in ECs from AAD model mice. Immunofluorescence and immunoblotting confirmed markedly increased Piezo1 protein in human and mouse AAD samples. Disturbed flow induced by abdominal aortic constriction plus Angiotensin II up-regulated Piezo1 expression and AAD incidence, whereas Piezo1 inhibition attenuated both. Moreover, EC-specific Piezo1 deficiency was sufficient to suppress β-aminopropionitrile- and Ang II-induced AAD formation in mice. Mechanistically, we discovered the disturbed shear stress-induced opening of the endothelial barrier via Piezo1 and subsequent Ca^2+^/MLC/PYK2/SRC-mediated activation of downstream signaling. In addition, in an Ang II-induced endothelial barrier dysfunction model, Piezo1 deficiency in ECs ameliorated Ang II-induced barrier dysfunction. Further study revealed that Piezo1 activation in ECs promoted the up-regulation of CCL2 in a STAT3-dependent manner and strongly promoted the chemotactic recruitment of monocytes/macrophages to the aorta. Importantly, blocking CCR2 reduced monocyte/macrophage recruitment and subsequently attenuated Piezo1 up-regulation-aggravated AAD formation. Transcriptome analysis of human AAD samples revealed a positive correlation between Piezo1 and inflammation of the aorta. We demonstrate that Piezo1 directly links mechanical sensing under disturbed flow to a pathogenic cascade of endothelial barrier dysfunction and inflammatory cell recruitment. Consequently, interventions targeting Piezo1 or the downstream STAT3-CCL2/CCR2 signaling hold therapeutic promise for AAD.

## Introduction

Aortic aneurysm and dissection (AAD) are life-threatening vascular diseases with a prevalence of 1.3% to 8% and high mortality due to the lack of effective therapies [1,2]. Aortic aneurysm is characterized by weakening and dilatation of the aorta, most commonly the abdominal segment [3]. Aortic dissection is an acute process in which a tear in the intimal layer allows blood to separate the intima-media from the adventitia [4]. Depending on the region involved, AAD is classified as thoracic aortic dissection (TAD) or abdominal aortic aneurysm (AAA). Patients are often asymptomatic and diagnosed incidentally during imaging or screening [5]. Despite surgical advances, therapies that limit AAD progression and rupture remain a priority. Risk factors for AAD include high LDL cholesterol, triglycerides, and inflammatory mediators and arterial hypertension, although the contribution from the local arterial microenvironment to AAD development remains unclear beyond these systemic factors.

Mechanically, AAD rupture occurs when hemodynamic loads exceed aortic wall strength [6]. At AAD sites, shear stress is altered by dilatation and thrombus, and aneurysmal flow often exhibits dominant channels with recirculation zones [7,8]. Hemodynamic forces also modulate inflammation and aortic diameter enlargement in AAD. Endothelial cells (ECs), which line the lumen and directly sense shear stress, are critical in the early stages of AAD [9].

Laminar versus disturbed flow elicits distinct EC signaling program [10,11]. Piezo1, a mechanosensitive ion channel located in the plasma membrane, permits Ca^2+^ influx upon mechanical activation [12–14]. It is primarily expressed in endothelial and smooth muscle cells and is essential for vascular mechanobiology. Reduced Piezo1 has been reported in thoracic aortic smooth cells in Marfan syndrome (MFS)-associated TAA [15], and endothelial Piezo1 loss exacerbates TAA formation [16]. Conversely, Piezo1 up-regulation in vascular smooth muscle cells appears detrimental in AAA [17]. These divergent roles in TAA versus AAA underscore the need to define Piezo1’s regulation and function in AAD.

Here, we identify Piezo1 as the most up-regulated mechanosensor in ECs from AAD model mice by single-cell RNA sequencing (scRNA-seq). Immunofluorescence and immunoblotting confirm marked Piezo1 elevation in human and mouse AAD. Disturbed flow induced by abdominal aortic constriction (AAC) combined with angiotensin II infusion increases Piezo1 expression and AAD incidence, whereas Piezo1 inhibition with GsMTx4 attenuates both. To define EC-intrinsic roles, we generated mice lacking Piezo1 in ECs (Piezo1^flox/flox^/Tek^Cre/+^ mice). Endothelial Piezo1 deficiency suppressed AAD development and adverse remodeling in vivo, and preserved endothelial barrier integrity. Mechanistically, Piezo1 activation disrupted the endothelial barrier via Ca^2+^-dependent signaling, up-regulated CCL2 in ECs, and promoted STAT3-dependent chemotactic recruitment of monocytes/macrophages, driving inflammation and vascular remodeling. Pharmacological blockade of the CCL2 receptor or STAT3 knockdown reduced leukocyte recruitment, prevented focal barrier opening and adhesion, and mitigated AAD formation. Transcriptome analysis of human AAD showed a positive correlation between Piezo1 expression and inflammatory signatures. Collectively, our findings demonstrate that disturbed flow promotes endothelial barrier dysfunction and AAD by activating Piezo1, which serves as the key hub connecting mechanosensing to barrier breakdown and immune cell recruitment, thereby highlighting Piezo1 and the downstream CCL2/CCR2-STAT3 axis as promising therapeutic targets.

## Results

### Piezo1 expression is markedly elevated in aortic intima in AAD patients and mice

**Fig. 1.**
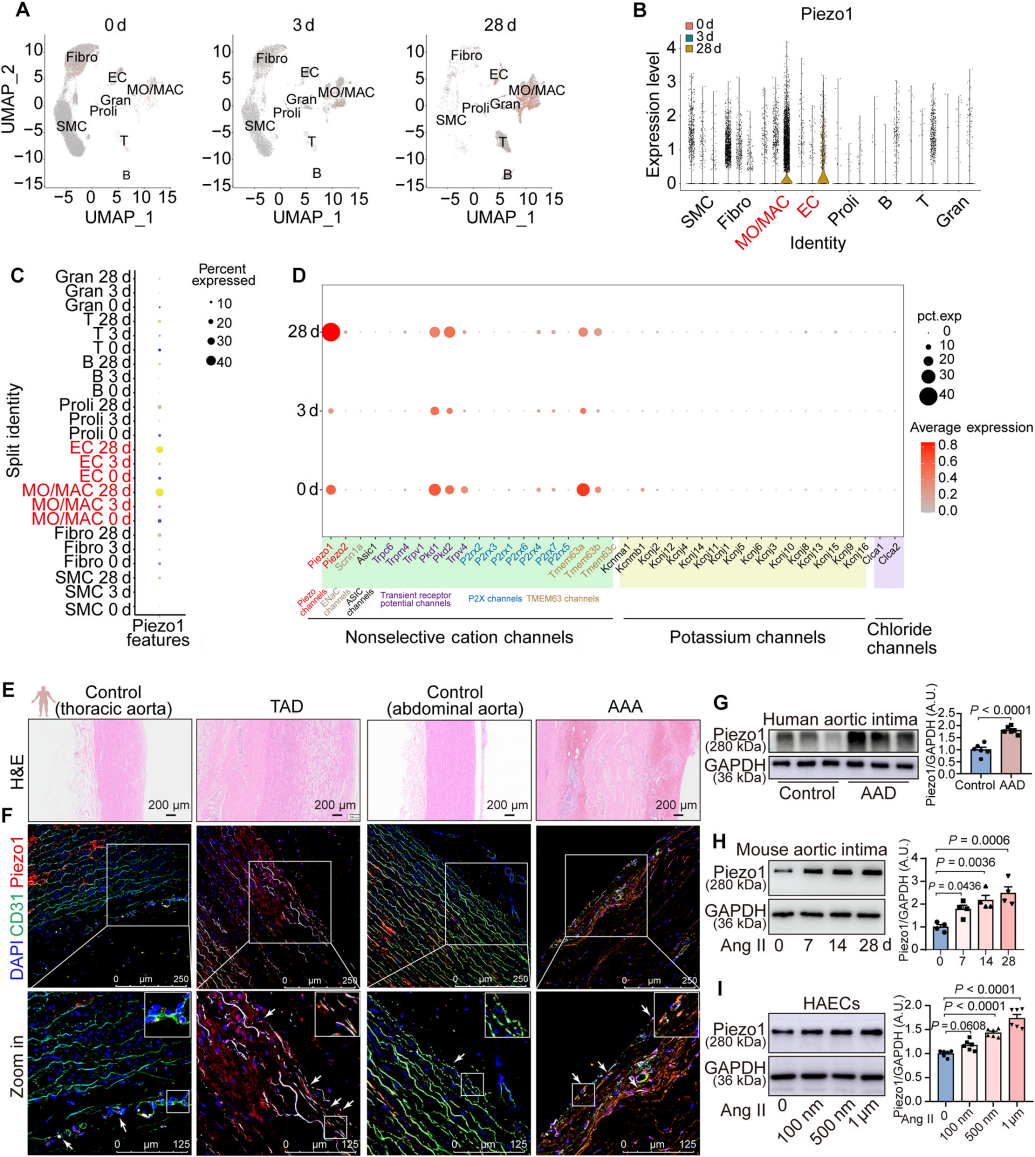
Piezo1 expression is elevated in patients and mice with aortic aneurysm and dissection (AAD). (A) The 8-week-old male ApoE^−/−^ mice were categorized into day 0 (*n* = 5 aortas), day 3 (*n* = 5 aortas), and day 28 groups (*n* = 5 aortas) based on the varying durations of Angiotensin II (Ang II) exposure. Full-length aortas were digested to obtain single-cell suspensions for sequencing with DNBelab C Series High-throughput Single-cell System (BGI-research). Uniform Manifold Approximation and Projection (UMAP) plot identified the expression and distribution of Piezo1 in each single cell of aortas from day 0 without Ang II exposure, or 3 and 28 days after Ang II exposure. (B) Violin plot visualizing the Piezo1 expression in each single cell of aortas from day 0 without Ang II exposure, or 3 and 28 days after Ang II exposure. (C) Dot plot representing the Piezo1 expression in each single cell. The size of the dot corresponds to the percentage of cells expressing Piezo1 (percent expressed) across day 0 without Ang II exposure, and 3 and 28 days after Ang II exposure. (D) Dot plot representing the mechanosensors expressions in endothelial cells (ECs). The color of the dot represents the mechanosensors’ expression levels (average expression), and the size of the dot corresponds to the percentage of cells expressing mechanosensors (percent expressed) across day 0 without Ang II exposure, and 3 and 28 days after Ang II exposure. (E) Representative hematoxylin and eosin (H&E) staining in the aortic tissues from TAD and AAA patients (*n* = 6 per group) and corresponding controls (*n* = 6 per group). Scale bar, 200 μm. (F) Representative images showing double immunostaining for Piezo1 (red) and CD31 (green) of the intima of aortic tissues from TAD and AAA patients (*n* = 6) and corresponding controls (*n* = 6). DAPI (blue) was used for nucleic acid labeling. Scale bar of low-magnification images, 250 μm. Scale bar of zoomed-in images, 125 μm. (G) Representative immunoblots and relative quantification analysis of Piezo1 protein in human control and AAD aortic intima tissues. Data are mean ± SEM. Unpaired Student *t* test (*n* = 6 per group). (H) Representative immunoblots and relative quantification analysis of Piezo1 protein in mouse aortic intima tissues from day 0 without Ang II exposure, or 7, 14, and 28 days after Ang II exposure. Data are mean ± SEM. One-way ANOVA with Dunnett’s post hoc analysis (*n* = 4 per group). (I) Representative immunoblots and relative quantification analysis of Piezo1 protein in human aortic endothelial cells (HAECs). Data are mean ± SEM. One-way ANOVA with Dunnett’s post hoc analysis (*n* = 6 per group).

To analyze the molecular profiles of individual cells during AAD development, we established an Ang II-induced AAD model in 8-week-old male ApoE^−/−^ mice and performed scRNA-seq on cells from abdominal aorta immediately before (day 0) and at 3 and 28 days following Ang II exposure. We found cellular heterogeneity, with an increased percentage of immune cells (monocyte/macrophage, T cells, B cells, and granulocytes) and a reduced percentage of nonimmune cells (vascular smooth muscle cells and fibroblasts) on days 3 and 28 after Ang II infusion (Fig. 1A). We first evaluated the expression of Piezo1 in individual cells and found that Piezo1 expression was markedly increased in ECs and monocytes/macrophages (Fig. 1A to C). Given that mechanical sensors can sense changes in blood flow shear stress in a timely manner during AAD, we compared expression of Piezo1 to that of several other channels with known functions in ECs and found that Piezo1 was the channel with the most prominent rise in expression (Fig. 1D). Immunohistochemical staining and Western blot revealed that Piezo1 levels were increased in aorta tissues from AAD patients and mice (Fig. S1A to E). We further verified that human AAD samples exhibited degraded elastic fibers (Fig. 1E) and that Piezo1 expression was increased in intima of human AAD samples, as shown by immunofluorescence staining and Western blot (Fig. 1F and G). Consistently, immunoblot assays showed that Piezo1 protein levels were markedly up-regulated in mouse aortic intima tissues as early as day 7 post-Ang II infusion (Fig. 1H). Moreover, Piezo1 levels were progressively increased in Ang II-treated human aortic endothelial cells (HAECs) (Fig. 1I). Collectively, these results from AAD patients and mice indicate that Piezo1 activation is an initiating event in the pathological cascade and may be involved in AAD pathogenesis.

### Piezo1 exaggerates disturbed shear stress-induced AAD development in abdominal aorta and Piezo1 inhibition ameliorates AAD formation

**Fig. 2.**
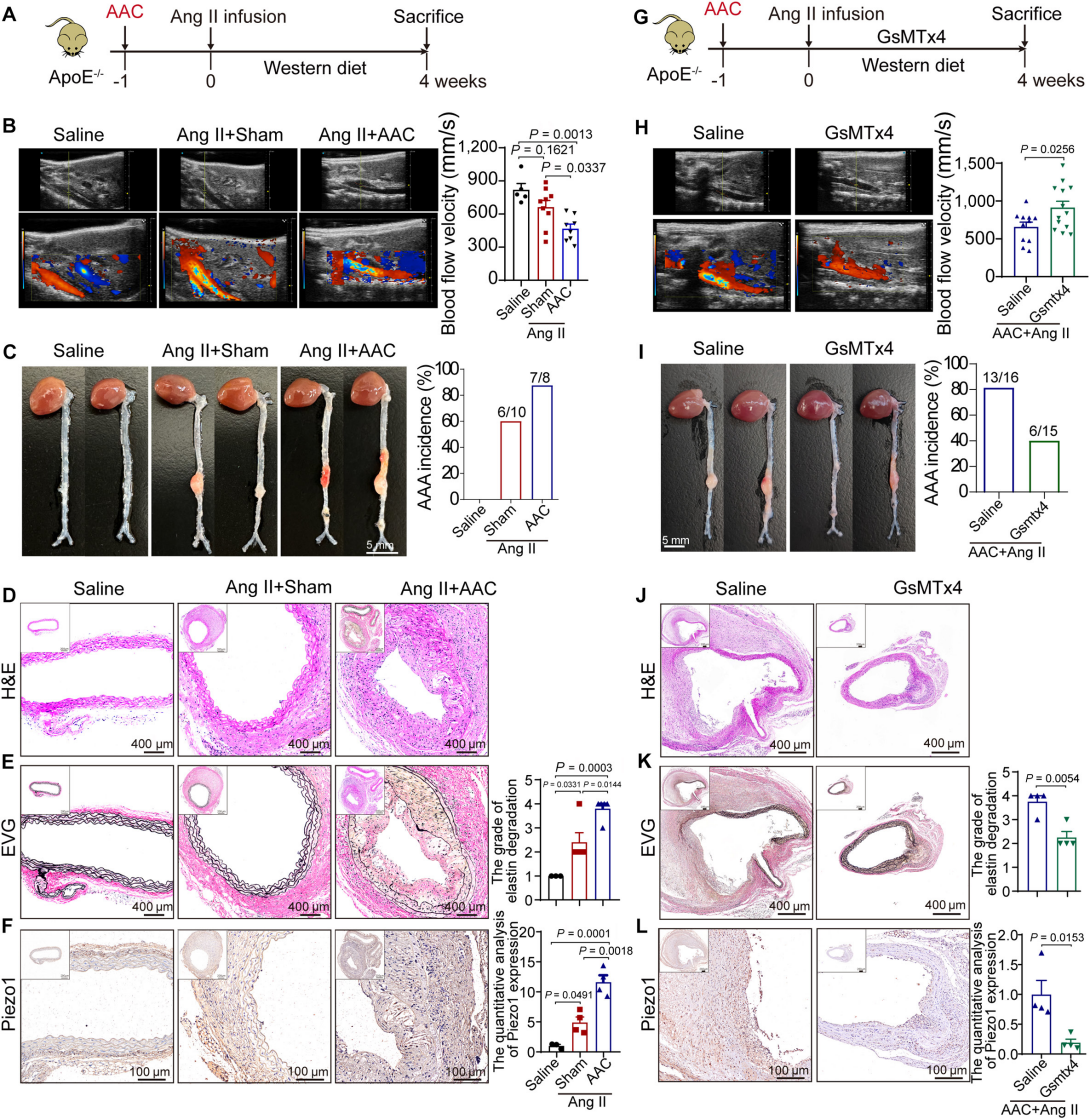
Disturbed shear stress in the abdominal aorta induces AAD formation via activating Piezo1 expression and inhibition of Piezo1 prevents AAD formation. (A) Schematic representation of study design. The 8-week-old male ApoE^−/−^ mice were constructed the abdominal aortic coarctation (AAC) model and were infused with Ang II or saline after 1 week (saline group, *n* = 5; Ang II+sham group, *n* = 10; Ang II+AAC group, *n* = 8). (B) Representative ultrasound images of the aorta and blood flow velocity across different groups. Data are mean ± SEM. One-way ANOVA with Dunnett’s post hoc analysis (saline group, *n* = 5; Ang II+sham group, *n* = 10; Ang II+AAC group, *n* = 8). (C) Representative images showing the morphology of the whole aorta showing the macroscopic characteristics of aneurysms and AAD incidence across different groups (saline group, *n* = 5; Ang II+sham group, *n* = 10; Ang II+AAC group, *n* = 8). Scale bar, 5 mm. (D) H&E staining of abdominal aorta from ApoE^−/−^ mice treated with saline, Ang II+sham, and Ang II+AAC. Scale bar, 400 μm. (E) EVG staining of abdominal aorta from ApoE^−/−^ mice treated with saline, Ang II+sham, and Ang II+AAC. Scale bar, 400 μm. Data are mean ± SEM. One-way ANOVA with Dunnett’s post hoc analysis (saline, *n* = 3; Ang II+sham, *n* = 5; Ang II+AAC, *n* = 5). (F) Representative Piezo1 immunohistochemical staining images and quantitative analysis of Piezo1 positive staining signals of aortas across different groups. Scale bar, 100 μm. Data are mean ± SEM. One-way ANOVA with Dunnett’s post hoc analysis (saline, *n* = 3; Ang II+sham, *n* = 4; Ang II+AAC, *n* = 4). (G) Schematic representation of study design. The 8-week-old male ApoE^−/−^ mice were constructed the AAC and Ang II model and treated with saline or the Piezo1 inhibitor GsMTx4 (10 mg/kg) via intraperitoneal injection every other day (saline group, *n* = 16; GsMTx4 group, *n* = 15). (H) Representative ultrasound images of the aorta and blood flow velocity between the 2 groups. Data are mean ± SEM. Unpaired Student *t* test (saline group, *n* = 11; GsMTx4 group, *n* = 13). (I) Representative images showing the morphology of the whole aorta showing the macroscopic characteristics of aneurysms and AAD incidence between 2 groups (saline group, *n* = 16; GsMTx4 group, *n* = 15). Scale bar, 5 mm. (J) H&E staining of aortic from ApoE^−/−^ mice treated with saline and GsMTx4. Scale bar, 400 μm. (K) EVG staining of aortic from ApoE^−/−^ mice treated with saline and GsMTx4. Scale bar, 400 μm. Data are mean ± SEM. Unpaired Student *t* test (*n* = 4 per group). (L) Representative Piezo1 immunohistochemical staining images and quantitative analysis of Piezo1 positive staining signals of aortas between 2 groups. Scale bar, 100 μm. Data are mean ± SEM. Unpaired Student *t* test (*n* = 4 per group).

Since Piezo1 serves as an ion channel that senses fluid stress and regulates cell function, we examined whether disturbed shear stress in abdominal aorta could modulate the expression of Piezo1 and AAD development and whether Piezo inhibition prevents AAD development. For this purpose, AAC was induced to disturb blood flow into abdominal aorta as previously described [18–21]. Besides, it was reported that Ang II-mediated vasoconstriction exacerbates luminal narrowing, potentially elevating shear stress at the stenotic site [22]. Therefore, to explore the impact of disturbed flow on AAD progression and involvement of Piezo1 in this mechanism, an abnormal shear stress model was established by combining Ang II administration with AAC surgery (Fig. 2A). After 5 weeks, aneurysmal aortas were dominated by low flow velocity and exhibited zones of recirculation in the Ang II plus sham group but not the saline group, while AAC aggravated these changes (Fig. 2B). Additional echocardiographic parameters pertaining to cardiac function are presented in Fig. S2A to E. Both ejection fraction and fractional shortening were markedly decreased in Ang II+sham and Ang II+AAC groups in comparison with the saline group. Left ventricular mass and systolic and diastolic left ventricular posterior wall thickness were markedly increased in the Ang II+sham and Ang II+AAC groups compared with the saline group. Cardiac function was comparable between the Ang II+sham and Ang II+AAC groups. AAC markedly heightened AAD incidence (Fig. 2C, AAD incidence: 60% in the Ang II+sham group versus 87.5% in the Ang II+AAC group). While other metabolic parameters were similar between the 2 groups, total cholesterol and LDL-C were specifically elevated in the Ang II+sham group compared to both the saline and Ang II+AAC groups (Fig. S2F to J). We further found that abdominal aorta presented much more severe elastin disruption and degradation in the Ang II+AAC group than in the Ang II+sham group, as manifested by hematoxylin and eosin (H&E) staining (Fig. 2D) and Verhoeff's Van Gieson (EVG) staining (Fig. 2E). We further observed that Piezo1 expression was markedly increased in the abdominal aortas of Ang II-treated mice, the effect of which was aggravated by AAC (Fig. 2F).

We next administered vehicle or the Piezo1 inhibitor GsMTx4 (10 mg/kg) via intraperitoneal injection every other day (Fig. 2G) [23]. After 5 weeks, the GsMTx4 group demonstrated a significant elevation in abdominal aortic blood flow velocity, approximating baseline hemodynamic levels observed in saline-treated controls (Fig. 2H). Compared with saline treatment, GsMTx4 treatment did not affect cardiac function or metabolic parameters (Fig. S2K to T). In the presence of Ang II, GsMTx4 treatment markedly lowered AAD incidence, compared with saline (Fig. 2I, AAD incidence: 40.0% in the GsMTx4 group versus 81.3% in the saline group). As evidenced by H&E staining and EVG staining, elastin disruption and degradation were alleviated in the GsMTx4 group compared with the saline group (Fig. 2J and K). Immunostaining analysis further revealed a significant down-regulation of Piezo1 expression (Fig. 2L). These results suggest that disturbed shear stress in the abdominal aorta caused by Ang II combined with AAC activates Piezo1 expression and promotes AAD development, whereas the inhibition of Piezo1 by GsMTx4 partially prevents these changes.

### Inhibition of Piezo1 ameliorates BAPN- and Ang II-induced AAD formation in vivo

We next verified these results by administering the Piezo1 inhibitor GsMTx4 to mice via intraperitoneal injection every other day. Notably, GsMTx4 did not overtly affect body weight (Fig. S3A) or metabolic parameters (Fig. S3B to F). After 4 weeks of β-aminopropionitrile (BAPN) treatment, GsMTx4 greatly decreased mortality (Fig. S4A) and TAD incidence (Fig. S4B and C, TAD incidence: 45.5% in the BAPN+GsMTx4 group versus 63.6% in the BAPN+Vehicle group). Echocardiographic assessment revealed a notable increase in the maximal diameter of the ascending aorta in the BAPN groups compared with the saline group, whereas inhibition of Piezo1 reversed these changes (Fig. S4D and E). As evidenced by H&E staining and EVG staining, the ascending aorta presented much more elastin disruption and degradation in the BAPN groups than in the saline group, whereas GsMTx4 intervention alleviated these changes (Fig. S4F and G).

In addition, GsMTx4 did not affect metabolic parameters (Fig. S3G to K) and mortality (Fig. S4H) but greatly decreased AAA incidence (Fig. S4I and J; AAD incidence: 36.4% in the Ang II+GsMTx4 group versus 63.6% in the Ang II+Vehicle group) after 4 weeks of Ang II treatment. Echocardiographic assessment in the GsMTx4 mouse group revealed a notable decrease in the maximal diameter of abdominal aorta (Fig. S4K and L), and H&E staining and EVG staining revealed much less elastin disruption and degradation in the GsMTx4 group than in the vehicle group (Fig. S4M and N). These results suggest that inhibition of Piezo1 by GsMTx4 partially attenuates BAPN- or Ang II-induced AAD development.

### Piezo1 deficiency in ECs ameliorates BAPN- and Ang II-induced AAD formation in mice

**Fig. 3.**
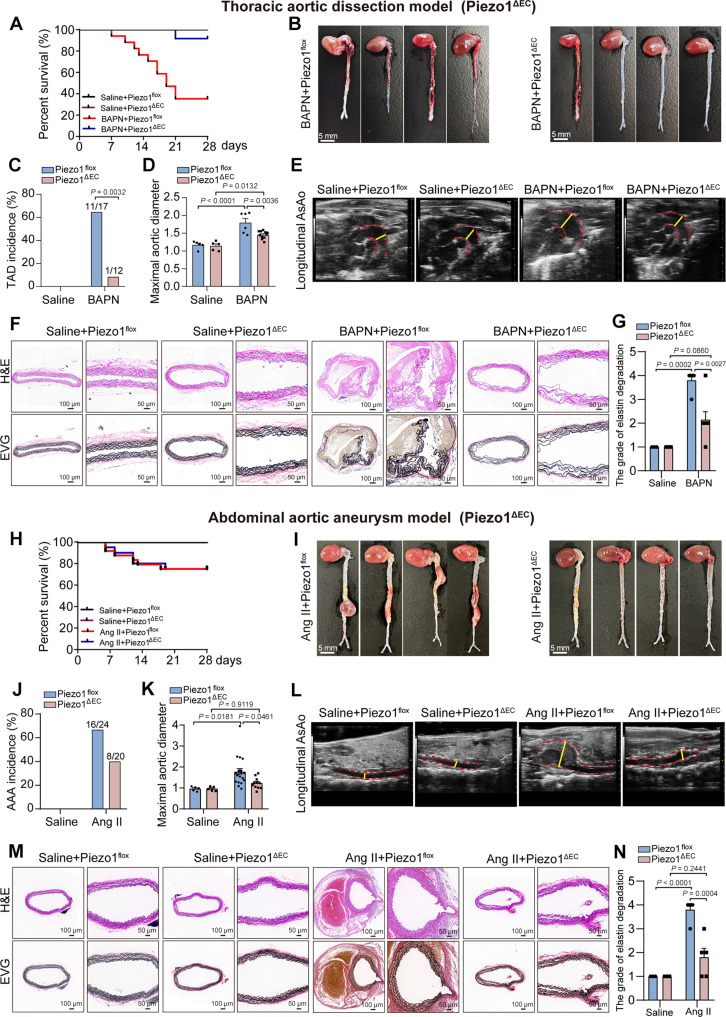
EC-specific Piezo1 deficiency represses BAPN- and Ang II-induced AAD formation in vivo*.* (A) The 4-week-old male Piezo1^flox^ mice and Piezo1^ΔEC^ mice were constructed the BAPN-induced TAD model. Survival curves in the indicated groups (saline+Piezo1^flox^, *n* = 5; saline+Piezo1^ΔEC^, *n* = 5; BAPN+Piezo1^flox^, *n* = 17; BAPN+Piezo1^ΔEC^, *n* = 12). (B) Representative macroscopic images between Piezo1^flox^ and Piezo1^ΔEC^ mice 28 days post-BAPN treatment. Scale bar, 5 mm. (C) TAD incidence in 4 male animal groups 28 days post-BAPN treatment (saline+Piezo1^flox^, *n* = 5; saline+Piezo1^ΔEC^, *n* = 5; BAPN+Piezo1^flox^, *n* = 17; BAPN+Piezo1^ΔEC^, *n* = 12). (D) Maximal aortic diameter in 4 male animal groups 28 days post-BAPN treatment. Data are presented as mean ± SEM. Two-way ANOVA with Dunnett’s post hoc analysis (saline+Piezo1^flox^, *n* = 5; saline+Piezo1^ΔEC^, *n* = 5; BAPN+Piezo1^flox^, *n* = 6; BAPN+Piezo1^ΔEC^, *n* = 11). (E) Representative ascending aortic ultrasound images in 4 animal groups at 4 weeks following BAPN infusion. (F) Representative H&E and EVG staining of mouse thoracic aorta in the indicated groups 28 days post-BAPN treatment. Low-magnification images in (F) show the entire vascular wall at the site of analysis; scale bar, 100 μm. Scale bar of high-magnification images in (F), 50 μm. (G) Grade of elastin degradation in the aortic wall. Data are presented as mean ± SEM. Two-way ANOVA with Dunnett’s post hoc analysis (saline+Piezo1^flox^, *n* = 3; saline+Piezo1^ΔEC^, *n* = 3; BAPN+Piezo1^flox^, *n* = 5; BAPN+Piezo1^ΔEC^, *n* = 7). (H) The 8-week-old male Piezo1^flox^ and Piezo1^ΔEC^ mice were injected with AAV8-PCSK9^D377Y^ before Ang II or saline infusion. Survival curves in the indicated groups (saline+Piezo1^flox^, *n* = 5; saline+Piezo1^ΔEC^, *n* = 5; Ang II+Piezo1^flox^, *n* = 24; and Ang II+Piezo1^ΔEC^, *n* = 20). (I) Representative macroscopic images between Piezo1^flox^ and Piezo1^ΔEC^ mice 28 days post-Ang II treatment. Scale bar, 5 mm. (J) AAA incidence in 2 groups 28 days post-Ang II treatment (saline+Piezo1^flox^, *n* = 5; saline+Piezo1^ΔEC^, *n* = 5; Ang II+Piezo1^flox^, *n* = 24; Ang II+Piezo1^ΔEC^, *n* = 20). (K) Maximal aortic diameter in 4 male animal groups 28 days post-Ang II treatment. Data are presented as mean ± SEM. Two-way ANOVA with Dunnett’s post hoc analysis (saline+Piezo1^flox^, *n* = 5; saline+Piezo1^ΔEC^, *n* = 5; Ang II+Piezo1^flox^, *n* = 19; Ang II+Piezo1^ΔEC^, *n* = 11). (L) Representative abdominal aorta ultrasound images in 4 male animal groups at 4 weeks following Ang II infusion. (M) Representative H&E and EVG staining of mouse abdominal aorta in the indicated groups 28 days post-Ang II treatment. Low-magnification images in (M) show the entire vascular wall at the site of analysis; scale bar, 100 μm. Scale bar of high-magnification images in (M), 50 μm. (N) Grade of elastin degradation in the aortic wall. Data are presented as mean ± SEM. Two-way ANOVA with Dunnett’s post hoc analysis (saline+Piezo1^flox^, *n* = 3; saline+Piezo1^ΔEC^, *n* = 3; Ang II+Piezo1^flox^, *n* = 5; Ang II+Piezo1^ΔEC^, *n* = 5).

Piezo1 expression was markedly up-regulated in ECs (Fig. 1B to D), and ECs are the first cells to be exposed to the shear stress generated by blood flow and play a vital role in the initial stage of AAD [24,25]. To further explore the role of endothelial Piezo1 in AAD development, we generated Piezo1^fl/fl^Tek^Cre+^ mice with conditional EC-specific Piezo1 knockout (Piezo1^ΔEC^, Fig. S5A and B). In our murine TAD model, mortality, TAD incidence, and the maximal diameter of the ascending aorta were markedly lower in Piezo1^ΔEC^ mice compared with Piezo1^flox^ mice (Fig. 3A to E). In addition, H&E staining and EVG staining revealed that Piezo1 deficiency in ECs notably ameliorated elastin disruption and degradation in the aortic wall on day 28 after TAD induction (Fig. 3F and G). In the Ang II-induced AAD model, elevated mortality, AAD incidence, and maximal diameter of abdominal aorta were attenuated by Piezo1 deficiency in ECs (Fig. 3H to L). Histological analysis revealed that Piezo1 deficiency in ECs decreased elastin disruption and degradation in the aortic wall on day 28 after AAD induction (Fig. 3M and N). Body weights and blood plasma lipid profiles of Piezo1^ΔEC^ mice and Piezo1^flox^ mice were comparable (Fig. S6A to J). Moreover, given the established role of Tek-Cre-mediated recombination in both ECs and myeloid cells, and to pinpoint the individual contributions of endothelial and immune cells, we performed bone marrow transplantation experiments and found that endothelial Piezo1 deficiency alone was sufficient to markedly attenuate AAA development, as evidenced by marked reductions in both incidence and maximal aortic diameter (Fig. S7). These results suggest that Piezo1 deficiency in ECs ameliorates BAPN- or Ang II-induced AAD development in mice.

### Disturbed shear stress impairs endothelial barrier function via Piezo1

**Fig. 4.**
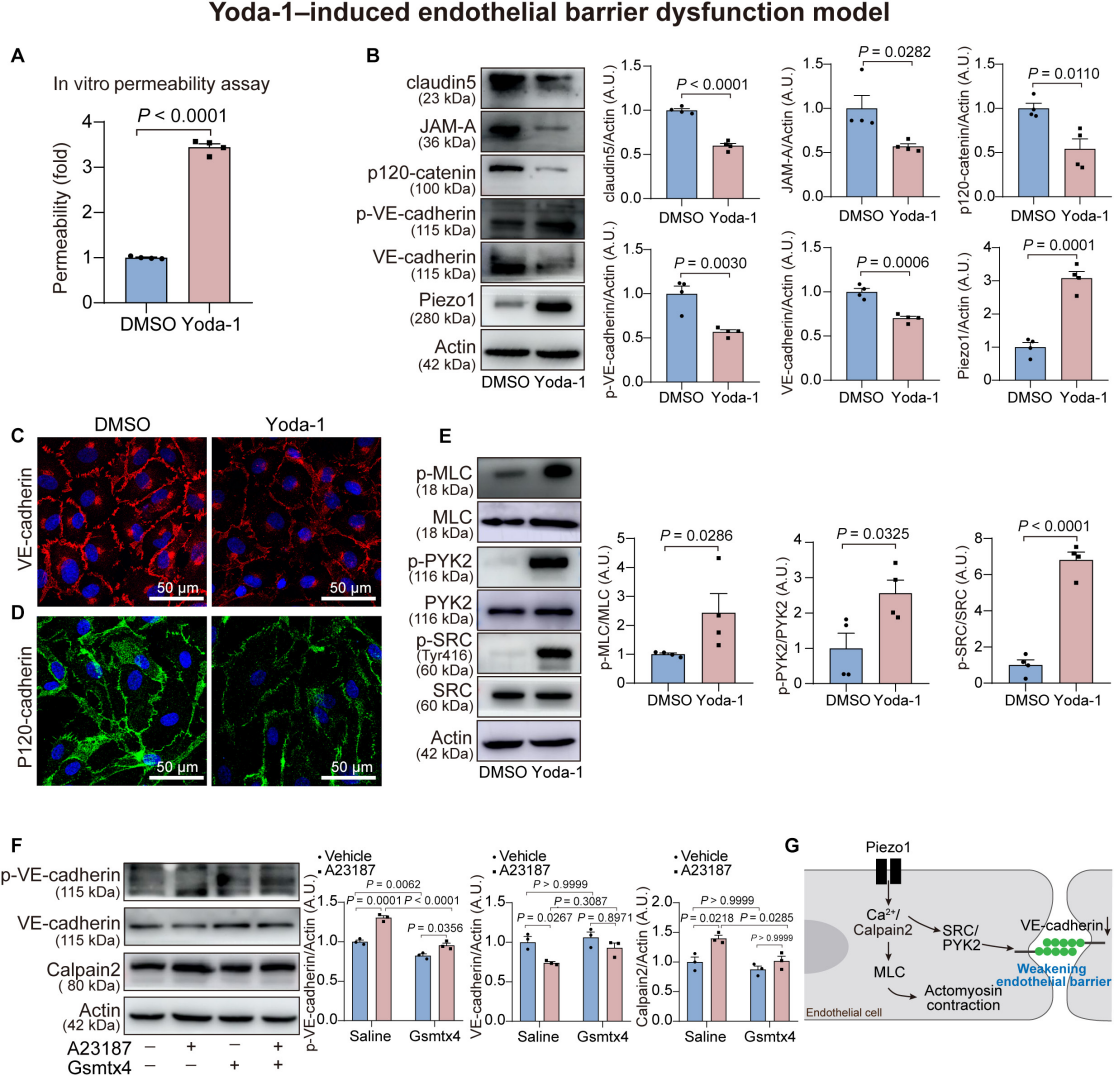
Yoda-1 impairs endothelial barrier function via Piezo1. (A) In vitro fluorescein isothiocyanate (FITC)-labeled dextran permeability assay for human aortic endothelial cells (HAECs) monolayers (Yoda-1 or DMSO) after 24 h of Ang II (10^−6^ M) treatment. Data are presented as mean ± SEM. Unpaired Student *t* test (Ang II+DMSO, Ang II+Yoda-1, *n* = 4 per group). (B) Representative immunoblots and quantification for claudin-5, JAM-A, p120-catenin, p-VE-cadherin, VE-cadherin, and Piezo1 in the indicated groups. Data are presented as mean ± SEM. Unpaired Student *t* test (*n* = 4 per group). (C) VE-cadherin-specific antibody staining (red; scale bars, 50 μm) and (D) p120-catenin-specific antibody staining (green; scale bars, 50 μm) of Yoda-1-treated HAECs. (E) Immunoblot analysis of total and phosphorylated MLC, PYK2, and SRC in lysates of HAECs treated with Yoda-1 or DMSO. Data are presented as mean ± SEM. Unpaired Student *t* test (DMSO, Yoda-1, *n* = 4 per group). (F) HAECs were stimulated with DMSO+vehicle, A23187+vehicle, DMSO+Gsmtx4, and A23187+Gsmtx4 for 24 h. Cells were harvested and p-VE-cadherin, VE-cadherin, and Calpain2 protein abundance was detected by Western blot. Two-way ANOVA with Dunnett’s post hoc analysis (*n* = 3 per group). (G) Schematic representation showing how fluid shear stress activate Piezo1 to induce downstream signaling events resulting in weakening endothelial barrier.

Earlier findings revealed that vascular endothelium acts as a regulated barrier and serves as the primary defense against hemodynamic and hormonal stimuli [26,27]. To confirm that Piezo1 was involved in the disturbed shear stress-induced opening of the endothelial barrier during AAD, we next tested whether the recently described specific Piezo1 activator Yoda-1 was able to induce effects similar to those mediated by Piezo1 in response to flow [28]. Similarly, Yoda-1 strongly increased HAECs permeability (Fig. 4A) and the protein level of p-VE-cadherin and decreased the protein levels of the endothelial barrier markers claudin-5, JAM-A, p120-catenin, and VE-cadherin (Fig. 4B). Consistent with these findings, immunofluorescence analysis revealed that Yoda-1 treatment reduced the expression of the barrier markers VE-cadherin (red) and p120-catenin (green) (Fig. 4C and D). It was reported that fluid shear stress increases endothelial plasma membrane tension to activate Piezo1. This leads to increases in Ca^2+^ levels and activation of downstream signaling events, including phosphorylation of myosin light chain (MLC), PYK2, and SRC, resulting in the opening of the endothelial barrier [29]. To determine whether mechanical cues generated by Piezo1 modulate the above signaling events to mediate the opening of the endothelial barrier during AAD, we assessed whether Yoda-1 indeed acts upstream of Ca^2+^ release and subsequent activation of MLC/PYK2/SRC and found that Yoda-1 strongly facilitated phosphorylation of MLC, PYK2, and SRC in HAECs (Fig. 4E). As shown in Fig. 4F, the calcium ionophore A23187 strongly up-regulated protein levels of the calcium-activated cysteine protease Calpain-2 and p-VE-cadherin, and such effects were strongly suppressed following Piezo1 inhibition (Fig. 4F). These data indicate the disturbed shear stress-induced opening of the endothelial barrier via Piezo1 and subsequent Ca^2+^/MLC/PYK2/SRC-mediated activation of downstream signaling (Fig. 4G).

### Piezo1 deficiency in ECs ameliorates Ang II-induced endothelial barrier dysfunction

**Fig. 5.**
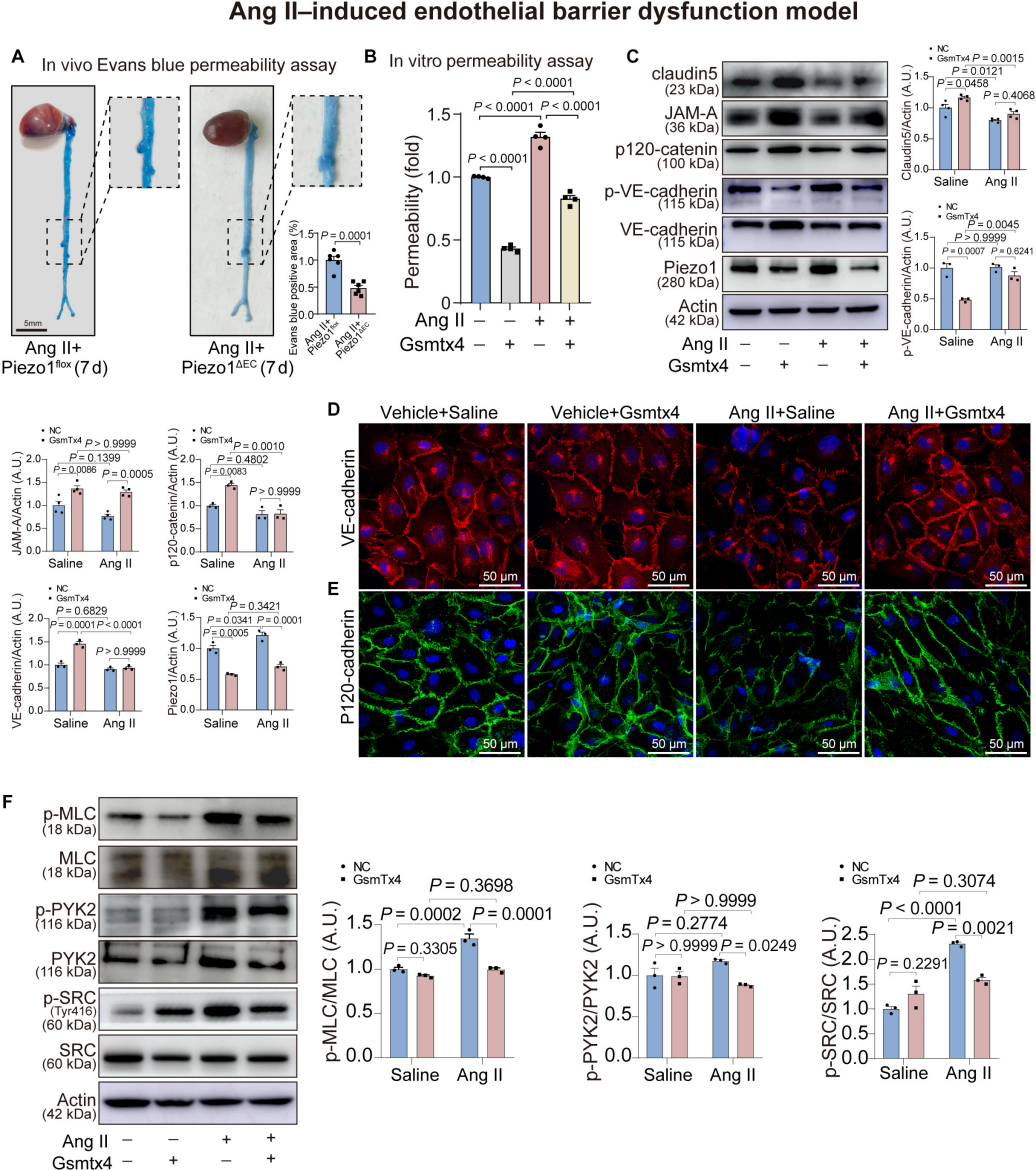
Piezo1 inhibition alleviates Ang II-induced endothelial barrier dysfunction in vitro. (A) Permeability of aortic intimal barrier (to Evans blue dye) and quantification of the Evans blue dye in Piezo1^flox^ and Piezo1^ΔEC^ mice 7 days post-Ang II treatment. Ang II+Piezo1^flox^, *n* = 6; and Ang II+Piezo1^ΔEC^, *n* = 6. Scale bars, 5 mm. (B) In vitro FITC-labeled dextran permeability assay for HAECs monolayers (Gsmtx4 or Vehicle) after 24 h of Ang II (10^−6^ M) treatment. Data are presented as mean ± SEM. Two-way ANOVA with Dunnett’s post hoc analysis (saline+Vehicle, Ang II+Vehicle, saline+Gsmtx4, Ang II+ Gsmtx4, *n* = 4 per group). (C) Representative immunoblots and quantification for claudin-5, JAM-A, p120-catenin, p-VE-cadherin, VE-cadherin, and Piezo1 in the indicated groups. Data are presented as mean ± SEM. Two-way ANOVA with Dunnett’s post hoc analysis (saline+Vehicle, Ang II+Vehicle, saline+Gsmtx4, Ang II+ Gsmtx4, *n* = 3 to 4 per group). (D) VE-cadherin-specific antibody staining (red; scale bars, 50 μm) and (E) p120-catenin-specific antibody staining (green; scale bars, 50 μm) of Ang II-transfected HAECs. (F) Immunoblot analysis of total and phosphorylated MLC, PYK2, and SRC in lysates of Ang II-induced HAECs treated with Gsmtx4 or Vehicle. Data are presented as mean ± SEM. Two-way ANOVA with Dunnett’s post hoc analysis (saline+Vehicle, Ang II+Vehicle, saline+Gsmtx4, Ang II+ Gsmtx4, *n* = 3 per group).

To further investigate the mechanism by which Piezo1 mediates endothelial barrier dysfunction under pathological hemodynamics, we compared endothelial barrier function in the early stage of AAA between Piezo1^ΔEC^ and Piezo1^flox^ mice. Using in vivo Evans blue permeability assay, Piezo1^ΔEC^ mice showed less appearance of Evans blue dye in the aorta compared with Piezo1^flox^ mice 7 days after Ang II treatment (Fig. 5A). Furthermore, we treated HAECs with the Piezo1 inhibitor GsMTx4. Given that disturbed shear stress is a key initiator of AAD, we employed Ang II stimulation as an in vitro model of such hemodynamic perturbation. We first determined the function of Piezo1 on endothelial permeability. As shown in Fig. 5B, Ang II challenge increased HAECs permeability, although such effect was largely attenuated by GsMTx4 (Fig. 5B). Western blot analysis revealed that GsMTx4 successfully reduced the level of Piezo1 and reversed Ang II-induced up-regulation of Piezo1 and p-VE-cadherin (Fig. 5C). In addition, Ang II-induced down-regulation of endothelial barrier markers claudin-5, JAM-A, p120-catenin, and VE-cadherin were all reversed by GsMTx4 (Fig. 5C). Consistently, immunofluorescence analysis further revealed that Ang II reduced expression of the barrier markers VE-cadherin (red) and p120-catenin (green), which was returned to near-normal levels by GsMTx4 (Fig. 5D and E). To test whether Piezo1 indeed acts upstream of MLC/PYK2/SRC in Ang II-induced endothelial barrier dysfunction model, Western blot analysis revealed that Ang II increased the phosphorylation of MLC, PYK2, and SRC in HAECs, whereas this effect was blunted by GsMTx4 treatment (Fig. 5F). These results suggest that Piezo1 induced MLC/SRC/PYK2 activation and resulted in the opening of the endothelial barrier.

### ECs secrete CCL2 upon Piezo1 activation and mediate the recruitment of monocytes to the aorta

**Fig. 6.**
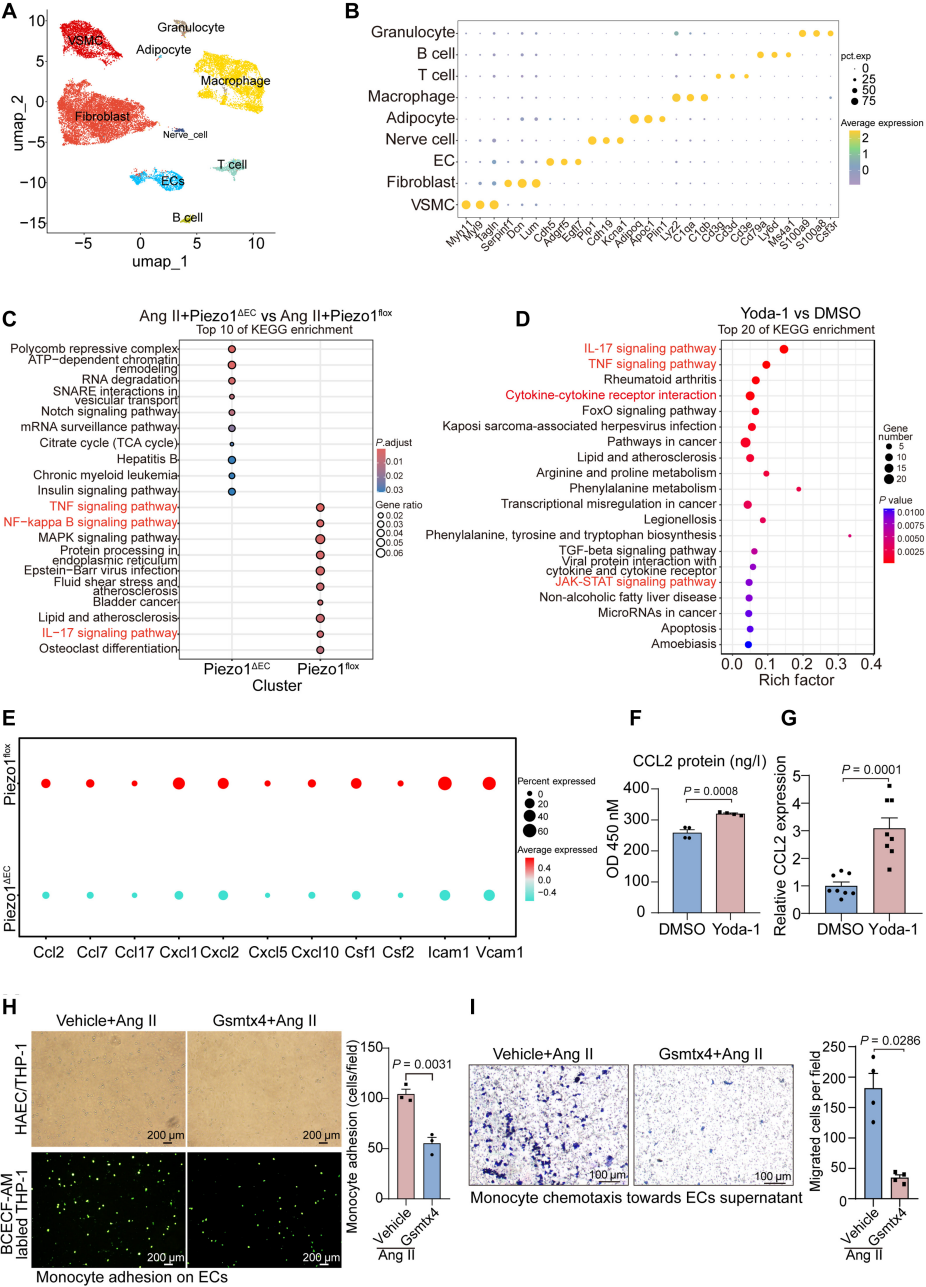
Piezo1 facilitates CCL2 chemoattraction and is essential for the monocyte/macrophage trans-endothelial migration. (A) Uniform Manifold Approximation and Projection (UMAP) plot identified 9 distinct cell populations of aortas from the Piezo1^flox^ and Piezo1^ΔEC^ mice treated with Ang II for 4 weeks (*n* = 5 per group). (B) A dot plot analysis indicating the relative expression of marker genes in distinct cell populations. Dot size reflects the percentage of cells expressing the selected gene in each population and dot color corresponds to expression level. (C) A list of top 10 enrichment pathways from KEGG enrichment analysis in ECs of aortas from the Piezo1^flox^ and Piezo1^ΔEC^ mice treated with Ang II. (D) Cultured HAECs were stimulated with DMSO and Yoda-1 (10 μM). These 2 group cells were collected for transcriptomic analysis (mRNA sequencing). Bubble plot showing the top 20 enriched KEGG pathways associated with the up-regulated differentially expressed genes in HAECs after Yoda-1 stimulation. (E) Expression of chemokines and cytokines in ECs from single-cell mRNA sequencing data. (F) Cultured HAECs were stimulated with DMSO and Yoda-1 (10 μM). Cellular supernatants were harvested and CCL2 protein expression was detected by ELISA. Data are presented as mean ± SEM. Unpaired Student *t* test (*n* = 4 per group). (G) Cultured HAECs were stimulated with DMSO and Yoda-1 (10 μM). Cells were harvested and chemokine CCL2 mRNA expression was detected by RT-qPCR. Data are presented as mean ± SEM. Unpaired Student *t* test (*n* = 8 per group). (H) Representative images and quantitative analysis of HAECs cocultured with THP-1 cells. Lightfield and [2′,7′-bis-(2-carboxyethyl)-5-(and-6)-carboxyfluorescein] acetoxymethyl ester (BCECF-AM)-labeled monocytes are shown. Scale bars, 200 μm. Data are presented as mean ± SEM. Unpaired Student *t* test (*n* = 3 per group). (I) Representative images and quantitative analysis of Transwell assays of HAECs. Cells migrating to the bottom were stained with crystal violet. Scale bars, 100 μm. Data are presented as mean ± SEM. Unpaired Student *t* test (*n* = 4 per group).

To further investigate the molecular mechanisms regulated by Piezo1 in ECs, we performed scRNA-seq on aortic tissues from the Piezo1^flox^ and Piezo1^ΔEC^ mice 28 days after Ang II infusion. Following enzymatic digestion, aortic cell suspensions with stringent quality control were sequenced using a 10x Genomics Single Cell System (CapitalBio Technology, Fig. S8A). Unsupervised Seurat-based clustering identified 9 distinct cell populations of aortas from the Piezo1^flox^ and Piezo1^ΔEC^ mice treated with Ang II for 4 weeks (Fig. 6A). All aortic cells were defined by 9 cell clusters shown by the dot plot and the top markers were identified based on genome alignment with CellRanger involving classic marker genes, such as Myh11, Myl9, and Tagln for vascular smooth muscle cell (VSMC); Serpinf1, Dcn, and Lum for fibroblasts; Cdh5, Adgrf5, and Egfl7 for ECs; Plp1, Cdh19, and Kcna1 for nerve cells; Adipoq, Apoc1, and Plin1 for adipocyte cells; Lyz2, C1qa, and C1qb for macrophages; Cd3g, Cd3d, and Cd3e for T cells; Cd79a, Ly6d, and Ms4a1 for B cells; S100a9, S100a8, and Csf3r for granulocytes (Fig. 6B). We then directed our focus toward ECs and analyzed differentially expressed genes (DEGs). Kyoto Encyclopedia of Genes and Genomes (KEGG) pathway analysis of marker genes expressed in ECs cluster revealed notable alterations in the TNF signaling pathway, NF-kappa B signaling pathway, and IL-17 signaling pathway, which were highly enriched in Piezo1^flox^ mice compared with Piezo1^ΔEC^ mice (Fig. 6C). Besides, we also employed mRNA-seq to profile the transcriptomes of ECs after Yoda-1 treatment (Fig. S9A). As shown in Fig. S9B, genes enriched in CXCR chemokine receptor binding and cytokine activity were mostly up-regulated after Yoda-1 treatment. The top 20 KEGG pathways associated with the up-regulated genes in HAECs following Yoda-1 stimulation were associated with IL-17 signaling, TNF signaling, and cytokine–cytokine receptor phagocytosis, suggesting the proinflammatory role of ECs following Piezo1 activation (Fig. 6D). Furthermore, we evaluated the specific expression of chemokines and cytokines among ECs using scRNA-seq data. As expected, genes including CC-motif chemokine ligand 2 (Ccl2), Ccl7, chemokine (C-X-C motif) ligand 1 (Cxcl1), Cxcl2, Icam1, and Vcam1 were up-regulated in Piezo1^flox^ mice compared with Piezo1^ΔEC^ mice (Fig. 6E). Next, we used enzyme-linked immunosorbent assay (ELISA) to measure CCL2 levels in the culture medium and found that compared with control cells, cells cultured in Yoda-1-containing medium exhibited higher CCL2 protein expression (Fig. 6F). Real-time quantitative PCR (RT-qPCR) analysis revealed that Yoda-1 increased the levels of the chemokine CCL2 (Fig. 6G). These results indicate that Piezo1 activation induced hyperactivation of EC inflammatory responses during AAD.

Recruitment of monocytes/macrophages from the bloodstream and their adhesion to ECs are important processes leading to vascular inflammation [30]. Since DEGs in ECs were predominantly involved in receptor ligand activity, signaling receptor activator activity, signaling receptor regulator activity, and signaling receptor binding in HAECs after Yoda-1 stimulation (Fig. S9C), we preferentially deciphered how Piezo1-mediated EC-monocyte/macrophage interactions contributed to AAD. We initially compared the quantity of TNF-α-challenged monocytes following GsMTx4 treatment and found that GsMTx4 did not affect monocyte proliferation (Fig. S10A). We further measured the ability of monocytes to adhere to ECs and found that, compared with control treatment, GsMTx4 treatment resulted in reduced adhesion of monocytes following Ang II insult (Fig. 6H). Additionally, the supernatant of ECs treated with a Piezo1 inhibitor strongly suppressed monocyte transmigration (Fig. 6I). We also examined macrophage transmigration and found that supernatant of TNF-α-stimulated ECs strongly promoted macrophage transmigration and that GsMTx4 reduced this effect (Fig. S10B). The supernatant of Yoda-1-stimulated ECs strongly facilitated macrophage transmigration (Fig. S10C). Immunofluorescent staining showed that the number of CD68^+^ macrophages was reduced in Piezo1^ΔEC^ mice and that the expression of several inflammatory cytokines and chemokines, including IL-1β, CCL2, TNF-α, and matrix metalloproteinase 2 (MMP2), exhibited a pronounced decrease in Piezo1^ΔEC^ mice (Fig. S11A). Collectively, these results suggested that Piezo1 activation perhaps enhanced macrophage inflammation in a paracrine manner.

Next, we investigated the effect of Piezo1 activation of ECs on the chemoattracted receptors for CCL2. CCR2 was the most down-regulated receptor in macrophages after Piezo1 inhibition in ECs (Fig. S12A). Further analysis revealed that the increases in protein levels of IL-6, TNF-α, IL-1β, and CCL2 by Yoda-1 were markedly alleviated by a CCR2 inhibitor in macrophages (Fig. S12B). Overall, these findings reveal that Piezo1 activation in ECs promotes CCL2 secretion and that activated monocytes/macrophages contain increased levels of CCR2, with the latter potentially recruiting more immune cells (Fig. S12C).

### Piezo1 regulates CCL2 and AAD formation through STAT3

**Fig. 7.**
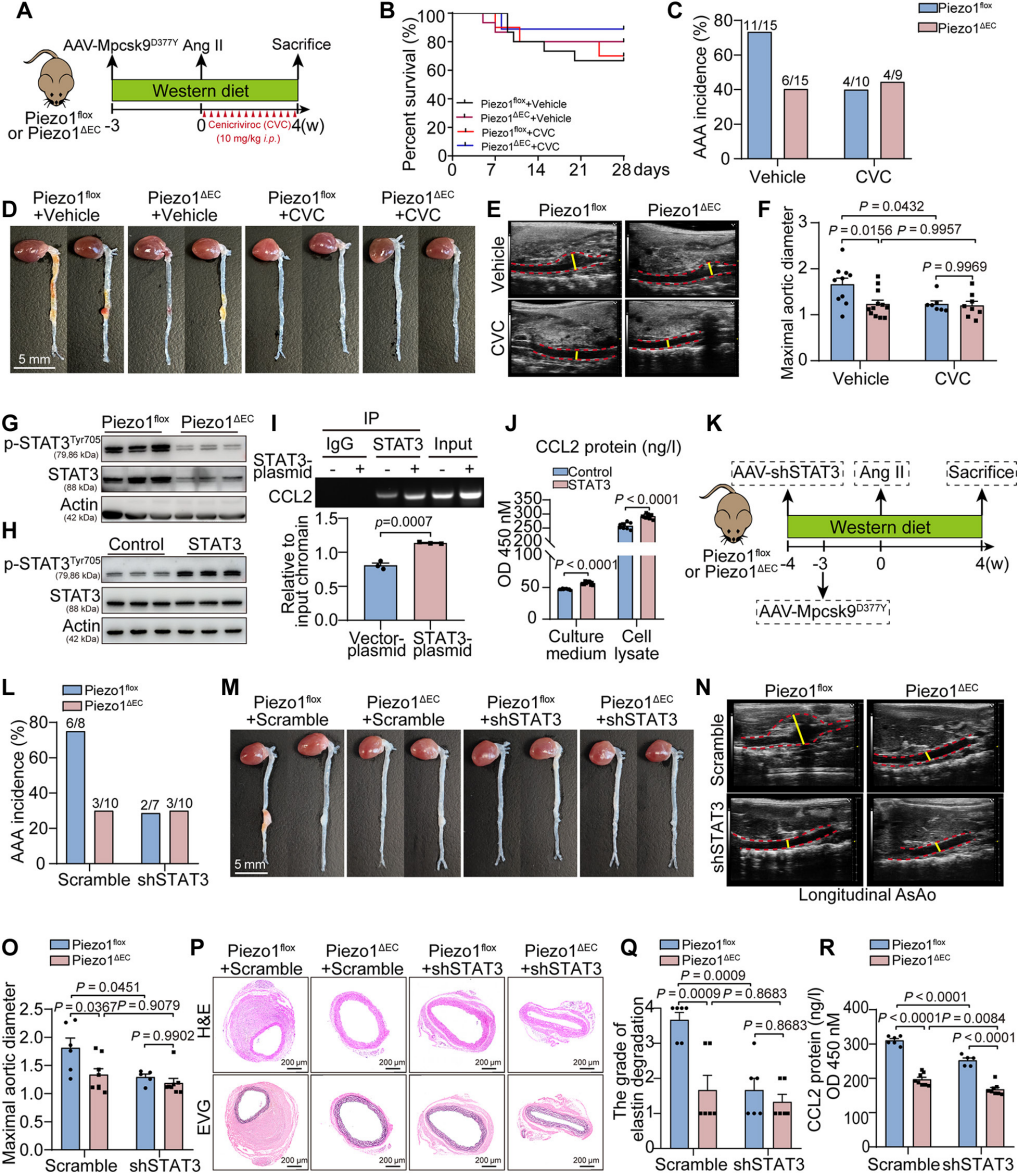
Piezo1 regulates CCL2 and AAD formation through STAT3, and treatment with CCR2 inhibitor or STAT3 knockdown reverses Piezo1-accelerated AAD progression. (A) Schematic representation of study design: The 8-week-old male Piezo1^flox^ and Piezo1^ΔEC^ mice were injected with AAV8-PCSK9^D377Y^ before Ang II infusion and were infused with cenicriviroc (CVC, 10 mg/kg) or Vehicle every other day. (B) Survival curves in 4 groups (Piezo1^flox^+Vehicle, *n* = 15; Piezo1^ΔEC^+Vehicle, *n* = 15; Piezo1^flox^+CVC, *n* = 10; Piezo1^ΔEC^+CVC, *n* = 9). (C) AAA incidence in 4 male animal groups 28 days post-Ang II treatment (Piezo1^flox^+Vehicle, *n* = 15; Piezo1^ΔEC^+Vehicle, *n* = 15; Piezo1^flox^+CVC, *n* = 10; Piezo1^ΔEC^+CVC, *n* = 9). (D) Representative macroscopic images between 4 male animal groups. Scale bar, 5 mm. (E) Representative abdominal aorta ultrasound images in 4 male animal groups at 4 weeks following Ang II infusion. (F) Maximal aortic diameter in 4 male animal groups 28 days post-Ang II treatment. Data are presented as mean ± SEM. Two-way ANOVA with Dunnett’s post hoc analysis (Piezo1^flox^+Vehicle, *n* = 10; Piezo1^ΔEC^+Vehicle, *n* = 12; Piezo1^flox^+CVC, *n* = 7; Piezo1^ΔEC^+CVC, *n* = 8). (G) Immunoblotting of p-STAT3 and STAT3 in the aortas of Piezo1^flox^ and Piezo1^ΔEC^ mice. (H) Immunoblotting of p-STAT3 and STAT3 in HAECs after STAT3 plasmid transfection. (I) Chromatin immunoprecipitation assay of STAT3 binding to CCL2 gene promoters in HAECs with or without STAT3 overexpression plasmid transfection. The data were presented as the means ± SEM. Student *t* test was used for statistical comparisons. *n* = 3 per group. (J) Cultured HAECs were stimulated with control and STAT3 plasmid. Cellular supernatants and cell lysates were harvested and CCL2 protein expression was detected by ELISA. Data are presented as mean ± SEM. Unpaired Student *t* test (*n* = 8 to 12 per group). (K) Schematic representation of study design: Eight-week-old male Piezo1^flox^ and Piezo1^ΔEC^ mice were first injected with AAV-shSTAT3 or AAV-Scramble. Following a 7-day incubation period, the animals subsequently received AAV8-PCSK9^D377Y^ viral vector delivery through tail vein injection for 3 consecutive weeks. The experimental protocol culminated with Ang II infusion via subcutaneously implanted osmotic minipumps to establish the AAA model. (L) AAA incidence in these animal groups 28 days post-Ang II treatment (Piezo1^flox^+Scramble, *n* = 8; Piezo1^ΔEC^+Scramble, *n* = 10; Piezo1^flox^+shSTAT3, *n* = 7; Piezo1^ΔEC^+shSTAT3, *n* = 10). (M) Representative macroscopic images between these animal groups 28 days post-Ang II treatment. Scale bar, 5 mm. (N) Representative abdominal aorta ultrasound images in these animal groups at 4 weeks following Ang II infusion. (O) Maximal aortic diameter in these animal groups 28 days post-Ang II treatment. Data are presented as mean ± SEM. Two-way ANOVA with Dunnett’s post hoc analysis (Piezo1^flox^+Scramble, *n* = 6; Piezo1^ΔEC^+Scramble, *n* = 8; Piezo1^flox^+shSTAT3, *n* = 5; Piezo1^ΔEC^+shSTAT3, *n* = 8). (P) Representative H&E and EVG staining of mouse abdominal aorta in the indicated groups 28 days post-Ang II treatment. Scale bar, 200 μm. (Q) Grade of elastin degradation in the aortic wall. Data are presented as mean ± SEM. Two-way ANOVA with Dunnett’s post hoc analysis (Piezo1^flox^+Scramble, *n* = 6; Piezo1^ΔEC^+Scramble, *n* = 6; Piezo1^flox^+shSTAT3, *n* = 6; Piezo1^ΔEC^+shSTAT3, *n* = 6). (R) Plasma CCL2 levels in these animal groups 28 days post-Ang II treatment. Data are presented as mean ± SEM. Two-way ANOVA with Dunnett’s post hoc analysis (Piezo1^flox^+Scramble, *n* = 6; Piezo1^ΔEC^+Scramble, *n* = 8; Piezo1^flox^+shSTAT3, *n* = 5; Piezo1^ΔEC^+shSTAT3, *n* = 8).

CCL2 is a pivotal inflammatory chemokine that regulates monocyte/macrophage trafficking and has been intensively studied as a potential target in many diseases, such as atherosclerosis and hepatocellular carcinoma [31,32]. Therefore, we examined the consequences of inhibiting CCL2 by blocking the migration of proinflammatory myeloid cells to the aorta using the dual C–C chemokine receptor type 2 and 5 antagonist cenicriviroc (CVC), which was developed and proven safe for the treatment of HIV infection and steatohepatitis [33]. Piezo1^ΔEC^ mice and Piezo1^flox^ mice infused with Ang II were administered with CVC every other day for 28 days (Fig. 7A). We found that CVC treatment reduced mortality (Fig. 7B), AAD incidence (Fig. 7C and D), and the maximal diameter of the abdominal aorta (Fig. 7E and F) in the aortic wall of mice on day 28 after AAD induction. Blood plasma lipid profiles of Piezo1^ΔEC^ mice and Piezo1^flox^ mice were comparable (Fig. S13A to E). These results demonstrate the potential of blocking the bone marrow-to-aorta migration of proinflammatory monocytes/macrophages as a strategy to prevent AAD.

We next investigated the molecular mechanism underlying Piezo1-induced CCL2 expression. STAT3 can lead to the production of CCL2, which promotes macrophage recruitment in human cancers [34–36]. In addition, a recent study reported that disturbed flow promotes endothelial inflammation and atherogenesis through the Piezo1-SRC-STAT3 pathway [37]. Our data also revealed that JAK-STAT signaling pathway was activated by Piezo1 (Fig. 6D). Thus, we suspected that Piezo1 can activate the transcription factor STAT3 to induce CCL2 transcription and increase macrophage recruitment during AAD development. Based on this prediction, we first assessed the level of STAT3 phosphorylation and found that Piezo1^ΔEC^ mice presented much lower STAT3 phosphorylation levels in the aorta than Piezo1^flox^ mice did (Fig. 7G). Notably, Western blot analysis revealed that the overexpression of STAT3 in ECs up-regulated STAT3 phosphorylation levels (Fig. 7H), and overexpression of STAT3 increased the STAT3 occupancy in CCL2 promoter based on chromatin immunoprecipitation followed by quantitative PCR (ChIP-qPCR) assay (Fig. 7I). Furthermore, ELISA revealed that CCL2 levels in the culture medium and cell lysate were increased in the STAT3 plasmid group compared to the control group (Fig. 7J). Next, we adopted the AAV1–recombinant intercellular adhesion molecule 2 (ICAM-2)–shSTAT3 system to selectively knockdown STAT3 in ECs on Piezo1^ΔEC^ mice and Piezo1^flox^ mice (Fig. 7K). As expected, shSTAT3 greatly lowered AAA incidence (Fig. 7L and M, AAA incidence: Piezo1^flox^+scramble, 75.0% versus Piezo1^ΔEC^+scramble, 30.0%; Piezo1^flox^+scramble, 75.0% versus Piezo1^flox^+shSTAT3, 28.6%; Piezo1^ΔEC^+scramble, 30.0% versus Piezo1^ΔEC^+shSTAT3, 30.0%) after 4 weeks of Ang II treatment. Echocardiographic assessment showed a notable decrease in the maximal diameters of the abdominal aorta after shSTAT3 treatment (Fig. 7N and O). As evidenced by H&E staining and EVG staining, the abdominal aorta presented much less elastin disruption and degradation in the shSTAT3 groups than in the scramble group (Fig. 7P and Q). ELISA of mouse blood showed that shSTAT3 greatly lowered the CCL2 expression (Fig. 7R). EC-specific STAT3 knockdown did not affect metabolic parameters 4 weeks after Ang II challenge (Fig. S13F to J). Altogether, these findings suggest that STAT3 signaling mediates, at least in part, Piezo1-induced production of CCL2, recruitment of monocytes/macrophages, and AAD formation.

### Piezo1 positively correlates with inflammation in patients with AAD

Next, we assessed whether Piezo1 gene expression was correlated with inflammation in patients with AAD. We analyzed mRNA expression in surgically resected media and adventitia from thrombus-free aspects of the abdominal aortic vessel wall of 34 patients with AAD and 13 organ donor controls (from GSE232911, Fig. S14A) [38]. We observed a positive correlation between Piezo1 expression with that of monocyte/macrophage markers CD68 and CD86, as well as T cell markers CD8A and CD4 in media but not in adventitia (Fig. S14B to E). Consistent with the correlation with inflammatory cell markers, we also observed a positive correlation with matrix metalloproteinases (MMP2 and MMP9, Fig. S14F and G) and intercellular adhesion molecule 1 (ICAM-1, Fig. S13H). Furthermore, we also observed a positive correlation between Piezo1 expression and vascular cell adhesion molecule 1 (VCAM-1) as well as cytokine expression—for example, CCL2, TNF, and CCL5. However, this was not significant according to the calculated *P* value (Fig. S14I to L).

Besides, we also assessed whether Piezo1 gene expression was correlated with inflammation in patients with type A aortic dissection. We analyzed mRNA expression in aortic media tissue samples obtained from 4 TAD patients and 4 organ donor controls (from GSE147026) [39]. The expression of these 7 genes (CD68, CD4, ICAM-1, VCAM-1, CCL2, TNF, and CCL5) also showed marked positive correlations with the expression of Piezo1 in TAD aorta (Fig. S15).

## Discussion

AAD, encompassing TAD and AAA with a high risk of rupture, carries a high rupture risk and is often asymptomatic and frequently detected incidentally [40]. Rupture constitutes a medical emergency [41], underscoring the need for effective pharmacological strategies to halt or reverse disease progression. Here, we show that EC-specific Piezo1 deficiency mitigates AAD development in vivo and suppresses shear stress-induced transendothelial monocyte migration. Inhibiting CCL2, a downstream effector of Piezo1, reproduced these protective effects. Notably, the Food and Drug Administration-approved CCR2/CCR5 antagonist CVC limited AAD enlargement, suggesting that pharmacologic targeting of the CCL2 axis with CVC may offer a feasible therapeutic approach for AAD.

Multiple murine AAD models recapitulate key features of human AAD. We employed 4 complementary systems to establish the pro-aneurysmal role of Piezo1: (a) Ang II-infused ApoE^−/−^ mice and (b) PCSK9/Ang II-treated C57BL/6J mice, both commonly used because they mirror several human AAD pathologies [40]; (c) BAPN administration, which inhibits lysyl oxidase, disrupts cross-linking of collagens and elastin, and precipitates thoracic aneurysm/dissection with frequent rupture; and (d) AAC with Ang II, which imposes disturbed hemodynamics and proinflammatory signaling. In our hands, disturbed shear stress in the abdominal aorta up-regulated Piezo1 and drove AAD formation. Endothelial Piezo1 deletion consistently attenuated disease across all models, namely, Ang II, PCSK9/Ang II, BAPN, and AAC/Ang II, demonstrating a robust, model-independent pathogenic role for Piezo1.

Aortic banding is a standard approach to induce pressure overload-mediated cardiomyocyte hypertrophy [21]. Compared with the widely used TAC model, AAC model produces chronic and progressive cardiac hypertrophy without overt systolic dysfunction at 1 month postsurgery [21]. Prior work shows that AAC-induced mechanical stress in the aortic value up-regulates Piezo1 and promotes aortic valvular calcification, whereas Piezo1 inhibition partially mitigates these effects [42]. In this study, we selected AAC because it more directly and severely disrupts abdominal aortic blood flow than other alternative methods. Relative to Ang II alone, AAC plus Ang II generated dominated flow channels with recirculation zones and further increased Piezo1 expression, indicating that disturbed abdominal aortic hemodynamics up-regulate Piezo1 and promote AAD. One limitation of this study is that we did not directly measure wall shear stress (WSS), which is the primary mechanical stimulus for endothelial mechanosensors including Piezo1, and thus WSS is not captured by any of our measurements. Additionally, we did not systematically interrogate other mechanical cues, including matrix stiffness and compressive stress, in ECs, which may also contribute to Piezo1-mediated AAD. Our focus was the Piezo1-dependent response to fluid shear in Ang II- and AAC-induced murine AAD. Furthermore, we observed higher total cholesterol and LDL-C in the Ang II-sham group compared with saline and Ang II-AAC groups, suggesting a potential protective metabolic adaptation to AAC whose mechanisms warrant further study.

Piezo1 appears to drive distinct mechanosensitive programs across murine AAD contexts. In AAA, Piezo1 is up-regulated in VSMCs, and its inhibition with GsMTx4 prevents aneurysm formation, as reported by Qian et al. [17]. Consistent with this, we observed increased aortic Piezo1 expression and found that inhibition of Piezo1 suppressed AAD. Extending these findings, we further show that hemodynamic cues activate endothelial Piezo1, promoting localized barrier opening and leukocyte diapedesis, a mechanism that may contribute to AAD pathogenesis. Piezo1 was up-regulated across all 3 aortic layers, most prominently in ECs and macrophages. Because ECs are the first sensors of shear stress, we focused on endothelial Piezo1 and confirmed that Ang II and disturbed flow up-regulate its expression. By contrast, Yang et al. [15] reported reduced Piezo1 in MFS aortas and reversal of TAA with the Piezo1 agonist Yoda-1. These discrepancies likely reflect divergent disease biology: MFS-related TAA arises from mutations in *FBN1* and features impaired VSMC force generation and mechanoresponsiveness [43,44]. We speculate that the reduced mechanosensitivity of Piezo1 in MFS renders channel activation protective (e.g., via SMAD2/3-TGFβ signaling) [15], whereas in abdominal or aortic arch ECs exposed to disturbed flow, Piezo1-mediated Ca^2+^ influx favors proinflammatory STAT3-CCL2 signaling and AAD. In our models (PCSK9/Ang II and BAPN), both global Piezo1 inhibition and EC-specific deletion lowered AAD incidence. Methodologically, while the published study used a Cadh5-CreERT2 line to delete Piezo1 specifically in ECs, our approach used a Tek-Cre mouse, which is also known to mediate recombination in hematopoietic cells. We propose that this methodological difference precisely underscores a distinct and complementary biological insight. The prior study elegantly defined the cell-autonomous requirement of endothelial Piezo1 in aortic homeostasis, using a model that selectively alters ECs. Our approach, while less restricted to a single lineage, allowed us to investigate the integrated, systemic function of the Piezo1 pathway within a more comprehensive physiological context relevant to disease pathogenesis. Together, these data support a context- and lineage-dependent role for Piezo1-pathogenic in ECs under disturbed flow, potentially compensatory in MFS VSMCs—arguing for site-specific Piezo1 targeting tailored to genetic background and clinical scenario.

In this study, we demonstrated that the Piezo1 activator Yoda-1 successfully mimics the physiological effects of flow-induced Piezo1 activation, leading to endothelial barrier compromise and subsequent activation of downstream signaling via Ca^2+^/MLC/PYK2/SRC. Conversely, pharmacological inhibition of Piezo1 with GsMTx4 significantly attenuated Ang II-induced endothelial barrier dysfunction in vitro. We acknowledge the inherent limitations of Yoda-1 and GsMTx4 regarding cell-type and target specificity. Recent evidence suggests that Yoda-1 may exert Piezo1-independent effects [45], while GsMTx4 is known to inhibit multiple mechanosensitive channels beyond Piezo1, including Transient Receptor Potential Canonical (TRPC) family members and volume-regulated anion channels [46]. To address these constraints and definitively establish the endothelial-autonomous role of Piezo1 in barrier function, we employed complementary genetic approaches. The striking phenotypic concordance between GsMTx4 treatment and EC-specific Piezo1 deletion, both of which attenuated endothelial permeability, preserved junctional proteins, and suppressed MLC/PYK2/SRC phosphorylation, provides rigorous genetic validation that these effects are mediated primarily through endothelial Piezo1. Consequently, our central conclusions regarding endothelial barrier regulation are primarily supported by our lineage-specific knockout models, with pharmacological agents serving as supportive, exploratory tools. Future studies using more selective Piezo1 modulators will further refine these findings with enhanced specificity.

Endothelial Piezo1 helps regulate barrier integrity across organs and may parallel mechanisms of vascular leakage in AAD. Under physiological conditions, Piezo1 supports blood–brain barrier (BBB) integrity by transducing mechanical cues in endothelium [47]. However, in pathological states such as intracranial hemorrhage or neurodegeneration, excessive mechanical stress or inflammatory stimuli can overactivate Piezo1, driving aberrant Ca^2+^ influx, endothelial dysfunction, tight junction disruption, and increased BBB permeability that facilitates entry of inflammatory mediators and pathogens [48]. Likewise, in the kidney, Piezo1 activation similarly exacerbates renal fibrosis [23,49], highlighting its context-dependent role in both vascular barrier regulation and fibrotic progression. The innovation of our work is to define a context-dependent mechanotransduction switch, rather than merely cataloging Piezo1 up- or down-regulation.

Piezo1 can modulate calpain activity via Ca^2+^ influx [13]. In our hands, elevating intracellular Ca^2+^ with A23187 increased levels of the Ca^2+^-activated cysteine protease calpain-2, an effect markedly blunted by Piezo1 inhibition. These data support a model in which endothelial Piezo1 initiates Ca^2+^-dependent signaling that engages MLC/PYK2/SRC pathways, linking mechanical cues to barrier dysfunction and inflammation.

Our findings position the present study at the intersection of mechanobiology, immunometabolism, and vascular pathophysiology. Hemodynamic stress gradients regulate inflammatory cell infiltration and atherosclerosis. Infiltrating leukocytes initiate wall injury that is then exacerbated by elevated wall stress, promoting AAD progression [9,50]. In our study, abnormal shear stress associated with AAD activated monocytes via Piezo1-dependent mechanotransduction. Shear stress-exposed monocytes showed enhanced adhesion to activated endothelium and up-regulated chemokine receptor CCR2 and inflammatory mediators (TNF-α, IL-1β, and IL-6, Fig. S11), providing a mechanistic link between local shear forces and amplified vascular inflammation. In the current study, we primarily utilized in vitro Transwell migration and adhesion assays to evaluate the role of Piezo1 in monocyte/macrophage recruitment. We acknowledge that we did not employ additional techniques—such as intravital microscopy to visualize dynamic cell trafficking in vivo, flow cytometry to quantify surface adhesion molecules (e.g., L-selectin, VLA-4, and LFA-1), or immunohistochemical analysis of tissue sections—to comprehensively assess this process. Consequently, while our data clearly demonstrate that endothelial Piezo1 activation facilitates a pro-recruitment environment, the precise in vivo dynamics of transendothelial migration remains to be fully elucidated, representing a limitation of the present study.

While the role of Piezo1 in endothelial inflammation during atherosclerosis is well established, its function in AAD endothelium remains underexplored. Disturbed flow can trigger Piezo1-mediated integrin activation and endothelial inflammation [51], and Piezo1 broadly contributes to immune activation, proliferation/migration, and cytokine production, connecting traditional and emerging atherosclerotic risks [52]. Given the marked Piezo1 elevation in monocyte/macrophages (Fig. 1A to C) and the importance of macrophage infiltration in AAD, we analyzed human aortic tissue and found that Piezo1 expression positively correlates with inflammatory markers in AAD versus donor controls. Collectively, beyond elucidating a key mechanism in AAD, our work implies a broader pathogenic paradigm relevant to atherosclerosis, thrombosis, and other inflammatory vasculopathies, in which Piezo1 acts as a central mechanosensitive node coordinating vascular immune-metabolic responses under dysregulated hemodynamics.

**Fig. 8.**
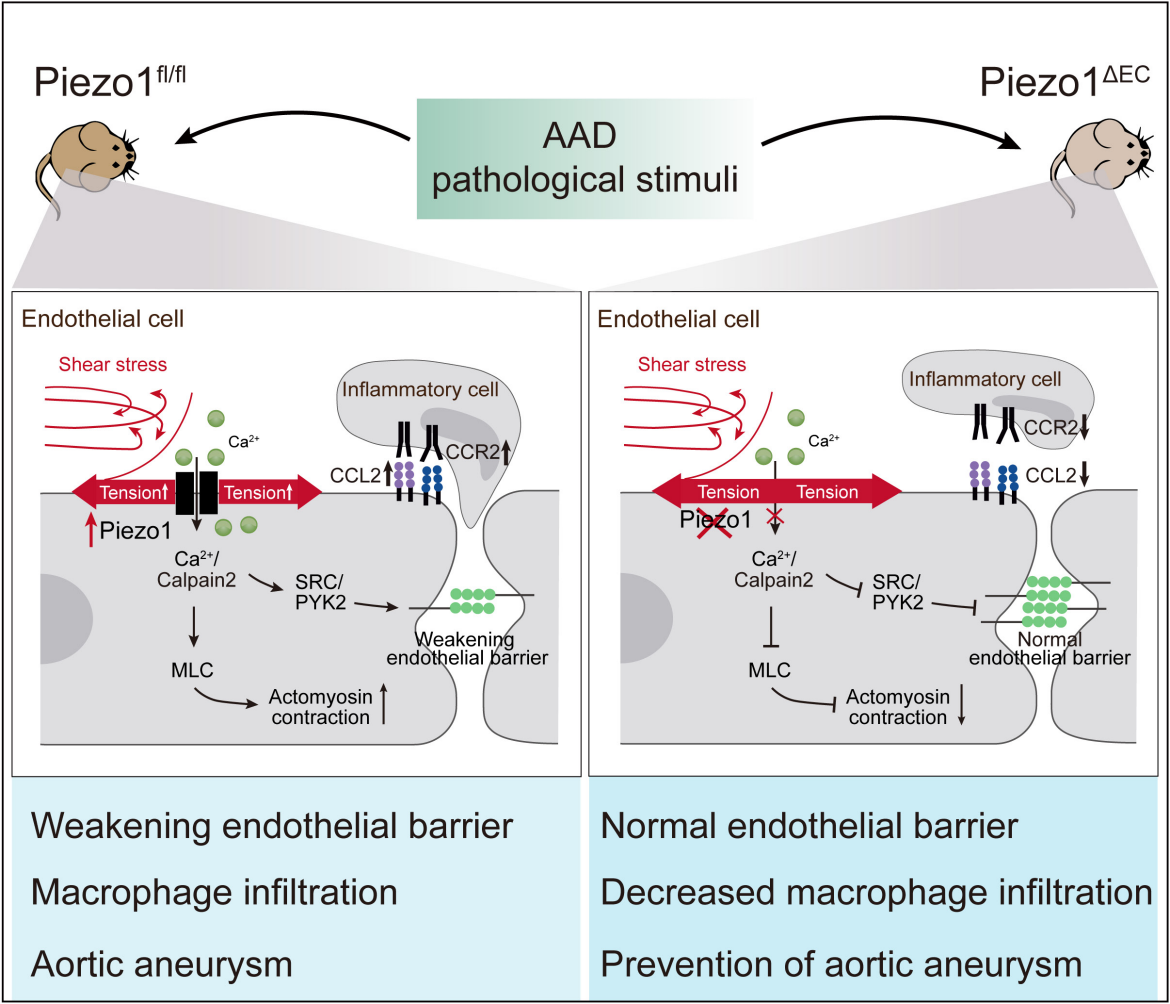
Schematic summary: under the AAD pathological environment, Piezo1 was up-regulated in aneurysmal endothelial cells. Endothelial-specific deficiency of Piezo1 was sufficient to suppress AAD formation. Moreover, Piezo1 deletion in ECs preserved endothelial barrier function through the Ca^2+^ and the downstream signaling events including phosphorylation of SRC, PYK2, and MLC. The localized opening of the endothelial barrier induced strong chemotactic recruitment of monocytes/macrophages to the aorta via CCL2–CCR2. Pharmacological therapy with cenicriviroc prevented and ameliorated Piezo1 up-regulation-induced AAD development.

In conclusion, the present study provides evidence that disturbed flow promotes endothelial barrier dysfunction and AAD through a Piezo1-dependent mechanism. Piezo1 activation in ECs engages Ca^2+^-dependent MLC/PYK2/SRC signaling, weakening junctional integrity and creating focal barrier openings. These sites facilitate monocyte adhesion and transendothelial migration, amplifying local inflammation and accelerating AAD pathology. Our findings integrate Piezo1-mediated mechanotransduction with EC-monocyte/macrophage crosstalk in the context of AAD (Fig. 8).

## Methods

A detailed methods section is available in the Supplemental Materials.

## Data Availability

Detailed materials and methods are presented in the Expanded Methods in the Supplemental Materials. All data that support the findings of this study are provided within the manuscript and will be made available to readers upon publication.

## Supplementary Materials

Supplementary 1 Materials and methods Tables S1 to S4 Figs. S1 to S15 Excel S1 to S3
