# Supplementary material for: Piezo1 Impairs Endothelial Barrier and Drives Aortic Aneurysm and Dissection via STAT3-Dependent Activation of the CCL2–CCR2 Axis
Source: Research (Wash D C). 2026 Jul 9;9:1351. doi: 10.34133/research.1351 (PMC13346660; doi:10.34133/research.1351)
Supplement: Supplementary 1 — Materials and methods Tables S1 to S4 Figs. S1 to S15 Excel S1 to S3 [file research.1351.f1.zip › R3--Supplemental material.docx]

**Piezo1 Impairs Endothelial Barrier and Drives Aortic Aneurysm and Dissection via STAT3-Dependent Activation of the CCL2–CCR2 Axis**

Running title：The Piezo1 mechanosensory pathway and aortic aneurysm and dissection

Kehui Yang^1,2,3,4,5†^, Yang Liu^1,2,3,4,5†^, Xiaoran Huang^6†^, Dehui Hou^1,2,3,4,5^, Han Du^1,2,3,4,5^, Jingwen Wang^1,2,3,4,5^, Hongwei Yue^1,2,3,4,5^, Yunyun Guo^1,2,3,4,5^, Sumei Cui^1,2,3,4,5^, Huidan Zhang^1,2,3,4,5^, Yijun Sun^1,2,3,4,5^, Xin Li^6*^, Jun Ren^7*^, Feng Xu^1,2,3,4,5*^, Yuguo Chen^1,2,3,4,5*^

^1^ Department of Emergency Medicine, Qilu Hospital, Shandong University, Jinan, China.

^2^ Shandong Provincial Clinical Research Center for Emergency and Critical Care Medicine, Institute of Emergency and Critical Care Medicine of Shandong University, Chest Pain Center, Qilu Hospital of Shandong University, Jinan, China

^3^ Medical and Pharmaceutical Basic Research Innovation Center of Emergency and Critical Care Medicine, China’s Ministry of Education, Shandong Provincial Engineering Laboratory for Emergency and Critical Care Medicine, Key Laboratory of Emergency and Critical Care Medicine of Shandong Province, Key Laboratory of Cardiopulmonary-Cerebral Resuscitation Research of Shandong Province, Qilu Hospital of Shandong University, Jinan, China

^4^ NMPA Key Laboratory for Clinical Research and Evaluation of Innovative Drug, Qilu Hospital of Shandong University, Jinan, China

^5^ National Key Laboratory for Innovation and Transformation of Luobing Theory; The Key Laboratory of Cardiovascular Remodeling and Function Research, Chinese Ministry of Education, Chinese National Health Commission and Chinese Academy of Medical Sciences, Qilu Hospital of Shandong University, Jinan, China

^6^ Department of Emergency Medicine, Guangdong Provincial People’s Hospital, Guangdong Academy of Medical Sciences, Southern Medical University, Guangzhou, China.

^7^ Department of Cardiology, Shanghai Institute of Cardiovascular Diseases, Zhongshan Hospital, Fudan University, Shanghai, China.

Correspondence to: Yuguo Chen, PhD, Email: chen919085@sdu.edu.cn. Feng Xu, PhD, Email: xufengsdu@126.com. Jun Ren, PhD, Email: ren.jun@zs-hospital.sh.cn. Xin Li, PhD, Email: [sylixin@scut.edu.cn](mailto:sylixin@scut.edu.cn)

^†^ K. Yang, Y. Liu, and X. Huang contributed equally.

* Y. Chen, F. Xu, J. Ren and X. Li contributed equally.

**This file includes:**

Supplementary Tables (Tables S1 to S4)

Supplemental figures and figure legends (Figures S1 to S15)

Supplementary methods

**Supplementary Tables**

**Table S1: Patient information for human aortic samples (n=15).**

| Variable | Sex | Race | Age (y) | Diagnosis | Smoking | Drinking | Hypertension | Diabetes Mellitus | Hyperlipidemia | Coronary artery disease |
| --- | --- | --- | --- | --- | --- | --- | --- | --- | --- | --- |
| TAA1 | Male | Yellow | 47 | Thoracic aortic aneurysm | Yes | Yes | Yes | Yes | No | No |
| TAA2 | Male | Yellow | 54 | Thoracic aortic aneurysm | No | No | Yes | No | No | No |
| TAA3 | Female | Yellow | 48 | Thoracic aortic aneurysm | No | No | No | No | No | No |
| TAD1 | Male | Yellow | 60 | Thoracic aortic dissection | Yes | No | Yes | No | No | Yes |
| TAD2 | Male | Yellow | 75 | thoracic aortic dissection | Yes | No | Yes | No | No | No |
| TAD3 | Female | Yellow | 47 | Thoracic aortic dissection | No | No | Yes | No | No | No |
| AAA1 | Male | Yellow | 60 | Abdominal aortic aneurysm | Yes | No | Yes | No | No | No |
| AAA2 | Male | Yellow | 59 | Abdominal aortic aneurysm | No | Yes | Yes | No | No | No |
| AAA3 | Female | Yellow | 48 | abdominal aortic aneurysm | No | No | No | No | No | No |
| AAA4 | Male | Yellow | 79 | Abdominal aortic aneurysm | No | Yes | No | No | No | Yes |
| AAA5 | Male | Yellow | 56 | Abdominal aortic aneurysm | No | No | Yes | No | No | No |
| AAA6 | Male | Yellow | 70 | abdominal aortic aneurysm | No | No | Yes | No | No | No |
| thoracic aortic control 1 | Male | Yellow | 54 | organ donor | No | Yes | Yes | No | No | No |
| thoracic aortic control 2 | Male | Yellow | 65 | organ donor | No | No | Yes | No | No | No |
| thoracic aortic control 3 | Male | Yellow | 49 | organ donor | No | No | No | Yes | No | No |
| abdominal aortic control 1 | Male | Yellow | 57 | organ donor | Yes | No | Yes | No | No | No |
| abdominal aortic control 2 | Male | Yellow | 60 | organ donor | No | Yes | No | No | No | No |
| abdominal aortic control 3 | Male | Yellow | 49 | organ donor | No | No | Yse | No | No | No |
| abdominal aortic control 4 | Male | Yellow | 37 | organ donor | No | No | Yes | No | No | No |
| abdominal aortic control 5 | Male | Yellow | 56 | organ donor | No | No | Yes | No | No | No |
| abdominal aortic control 6 | Male | Yellow | 40 | organ donor | No | No | Yes | No | No | No |

**Table S2: Summary of animal models**

| Models | Groups | Week age | Sex | Genetic background | AAD incidence |
| --- | --- | --- | --- | --- | --- |
| Ang II+AAC  model | Saline  (n=5) | 8 | Male | ApoE^−/−^ | 0 |
|  | Ang II+Sham  (n=10) | 8 | Male | ApoE^−/−^ | 60% |
|  | Ang II+ AAC  (n=8) | 8 | Male | ApoE^−/−^ | 87.5% |
| Ang II+AAC+GsMTx4  model | Ang II+AAC+Vehicle  (n=16) | 8 | Male | ApoE^−/−^ | 81.3% |
|  | Ang II+AAC+GsMTx4  (n=15) | 8 | Male | ApoE^−/−^ | 40% |
| BAPN+Piezo1^ΔEC^  model | Saline+Piezo1^ﬂox^  (n=5) | 4 | Male | Piezo1^ﬂox^ | 0 |
|  | Saline+Piezo1^ΔEC^  (n=5) | 4 | Male | Piezo1^ΔEC^ | 0 |
|  | BAPN+Piezo1^ﬂox^  (n=17) | 4 | Male | Piezo1^ﬂox^ | 64.7% |
|  | BAPN+Piezo1^ΔEC^  (n=12) | 4 | Male | Piezo1^ΔEC^ | 8.3% |
| Ang II+Piezo1^ΔEC^  model | Saline+Piezo1^ﬂox^  (n=5) | 8 | Male | Piezo1^ﬂox^ | 0 |
|  | Saline+Piezo1^ΔEC^  (n=5) | 8 | Male | Piezo1^ΔEC^ | 0 |
|  | Ang II+Piezo1^ﬂox^  (n=24) | 8 | Male | Piezo1^ﬂox^ | 66.7% |
|  | Ang II+Piezo1^ΔEC^  (n=20) | 8 | Male | Piezo1^ΔEC^ | 40% |
| BAPN+GsMTx4  model | Saline+Vehicle  (n=5) | 4 | Male | C57BL/6J | 0 |
|  | Saline+GsMTx4  (n=5) | 4 | Male | C57BL/6J | 0 |
|  | BAPN+Vehicle  (n=11) | 4 | Male | C57BL/6J | 63.6% |
|  | BAPN+GsMTx4  (n=11) | 4 | Male | C57BL/6J | 45.5% |
| Ang II+GsMTx4  model | Ang II+Vehicle  (n=11) | 8 | Male | ApoE^−/−^ | 63.6% |
|  | Ang II+GsMTx4  (n=11) | 8 | Male | ApoE^−/−^ | 36.4% |

**Table S3: Primers and shRNA sequences used in the study.**

| Gene name | Forward primers | Reverse primers |
| --- | --- | --- |
| Homo-CCL2 | GCCTCCAGCATGAAAGTCTC | GGCATTGATTGCATCTGGCT |
| Homo-VCAM1 | AGATGGCGCCTATACCATCC | TAGAGCACGAGAAGCTCAGG |
| Homo-β-actin | GGAAATCGTGCGTGACATTAA | AGGAAGGAAGGCTGGAAGAG |
| Mus-Vcam1 | GGAAATGCCACCCTCACCTT | GATCCGGGGGAGATGTCAAC |
| Mus-Vcam1 | CTGGGAAGCTGGAACGAAGT | GCCAAACACTTGACCGTGAC |
| Mus-ICAM1(CD54) | CAAAGCTCGACACCCCTGAC | GTTTGTGCTCTCCTGGGTCG |
| Mus-Cd68 | ACTTCGGGCCATGTTTCTCT | GGGGCTGGTAGGTTGATTGT |
| Mus-IL1β | GTGTCTTTCCCGTGGACCTT | AATGGGAACGTCACACACCA |
| Mus-IL6 | CTTCTTGGGACTGATGCTGGT | CTCTGTGAAGTCTCCTCTCCG |
| Mus-MCP1 | ACCACCTCAAGCACTTCTGT | TAAGGCATCACAGTCCGAGT |
| Mus-Tnfα | CGGGCAGGTCTACTTTGGAG | ACCCTGAGCCATAATCCCCT |
| Mus-Arg1 | TGACGGACTGGACCCATCTT | GGCTTGTGATTACCCTCCCG |
| Mus-CCR2 | CCAACGAGAGCGGTGAAGAA | TGAACACCAGCGAGTAGAGC |
| Mus-CCR3 | CCGGTGATCTACGCCTTTGT | AGCAAGTGCCTGTGGAAGAA |
| Mus-CCR5 | TTACTGTCCCCTTCTGGGCT | AAGCAAACACAGCATGGACG |
| Mus-CCR7 | ATGGTGATCGGCTTTCTGGT | CCAGGACCACCCCATTGTAG |
| Mus-CCR8 | GTTGTCCATGCCGTGTATGC | CGGTTAGCCATACTGCCAGG |
| Mus-LAF-1 | ATCCTGACTCCATTCGCTGC | GCGTCACTTTTTGTGGGGAC |
| Mus-β-actin | TGAGCTGCGTTTTACACCCT | TTTGGGGGATGTTTGCTCCA |
|  | Sequence (5' → 3') | |
| STAT3 shRNA | GCCTCAAGATTGACCTAGA | |

**Table S4: Details of antibodies and reagents applied in the study**

| Antibody | Company | Catalog No. |
| --- | --- | --- |
| Calpain 2 | Abcam | ab126600 |
| CD31 | Abcam | ab7388/ab9498 |
| CD68 | Abcam | ab955/ab53444 |
| claudin-5 | Abcam | ab131259 |
| IL-1β | Cell Signaling Technology | #12242S |
| IL-6 | Abcam | ab290735 |
| JAM-A | Abcam | ab269948 |
| MLC | Cell Signaling Technology | #3672 |
| Piezo1 | Abcam | ab259949 |
| Piezo1 | Proteintech | 15939-1-AP |
| p-MLC | Cell Signaling Technology | #3675 |
| p-SRC | Cell Signaling Technology | #6943 |
| PYK2 | Cell Signaling Technology | #3292 |
| p-PYK2 | Cell Signaling Technology | #3291 |
| p120-catenin | Santa Cruz Biotechnology | sc-23873 |
| SRC | Cell Signaling Technology | #2109 |
| TNF-α | Santa Cruz Biotechnology | sc-52746 |
| VE-cadherin | Abcam | ab33168 |
| β-actin | Cell Signaling Technology | #3700 |
| Reagent | Company | Catalog No. |
| Angiotensin II human | MCE | HY-13948 |
| BAPN | Sigma | A3134 |
| Calcimycin | MCE | 52665-69-7 |
| Cenicriviroc | Macklin | C878812 |
| Crystal Violet Staining Solution | Beyotime | C0121 |
| GsMTx4 | Selleck | p1205 |
| PD123319 | MCE | HY-10259 |
| Telmisartan | GlpBio | GC15023 |
| Yoda-1 | Selleck | S6678 |

**Supplemental Figures and Figure Legends**

**Figure S1**


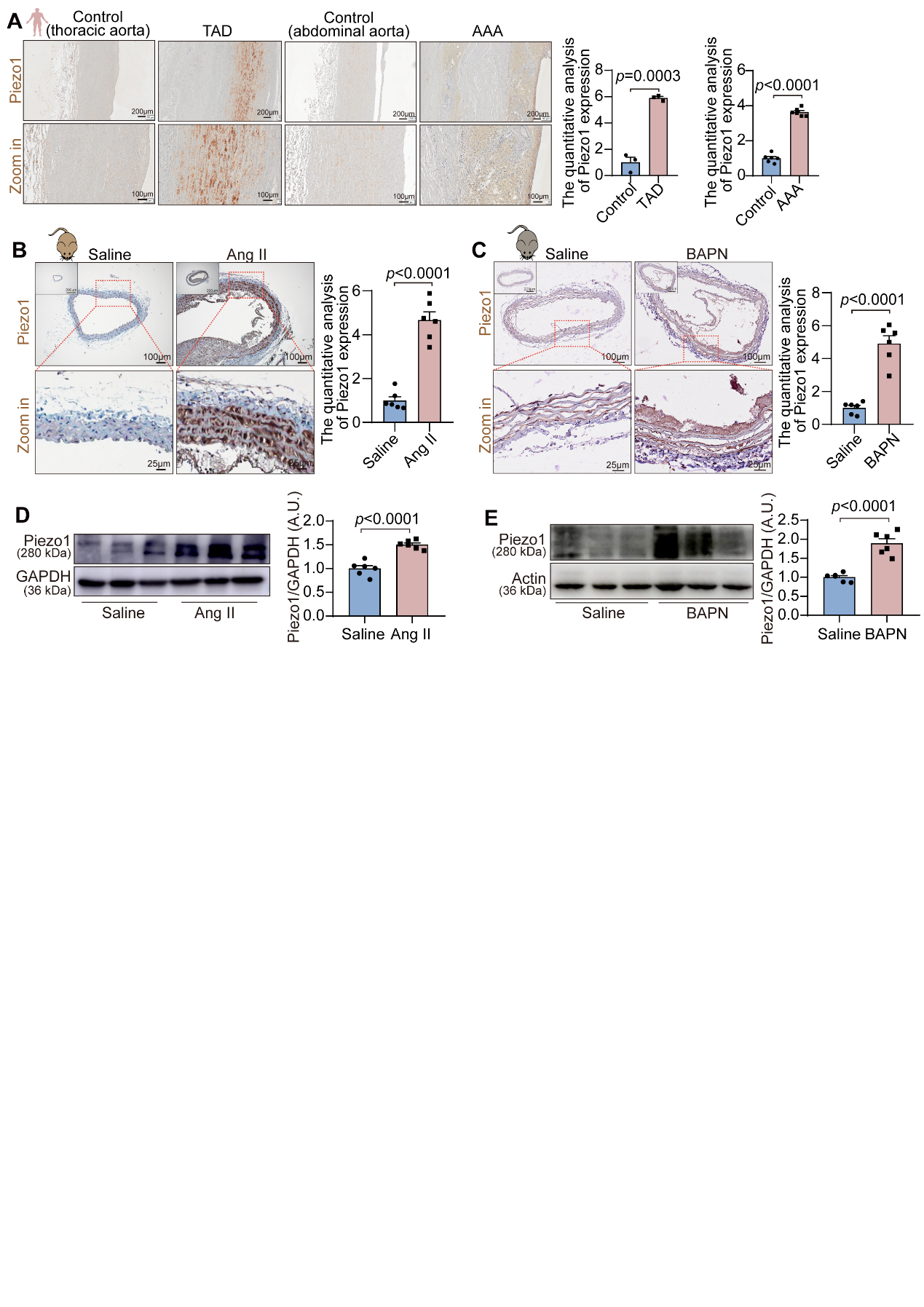


**Figure S1. Increased expression of Piezo1 in AAD. (A)** Representative Piezo1 immunohistochemical staining images and quantitative analysis of Piezo1 positive staining signals in the intima of aortic tissues from AAD patients and controls. Scale bar of low-magnification images, 200 μm. Scale bar of intima zoom in images, 100 μm. Data are mean±SEM. unpaired Student’s t-test (thoracic aorta control, TAA, TAD, n=3 per group; abdominal aorta control, AAA, n=6 per group). **(B)** Representative Piezo1 immunohistochemical staining images and quantitative analysis of Piezo1 positive staining signals in mouse control and angiotensin II (Ang II)–induced abdominal aortic aneurysm (AAA) aorta tissues (n=6 per group). Scale bar of low-magnification images, 100 μm. Scale bar of zoom in images, 25 μm. Data are mean±SEM. Unpaired Student’s t-test (n=3 per group). **(C)** Representative Piezo1 immunohistochemical staining images and quantitative analysis of Piezo1 positive staining signals in mouse control and 3-aminopropionitrile fumarate (BAPN)-induced thoracic aortic dissection (TAD) aorta tissue. Scale bar of low-magnification images, 100 μm. Scale bar of zoom in images, 25 μm. Data are mean±SEM. Unpaired Student’s t-test (n=6 per group). (**D)** Representative immunoblots and relative quantification analysis of Piezo1 protein in mouse control and Ang II–induced AAA aorta tissues. Data are mean±SEM. unpaired Student’s t-test (n=6 per group). (**E)** Representative immunoblots and relative quantification analysis of Piezo1 protein in mouse control and BAPN-induced TAD aorta tissues. Data are mean±SEM. Unpaired Student’s t-test (n=6 per group).

**Figure S2**


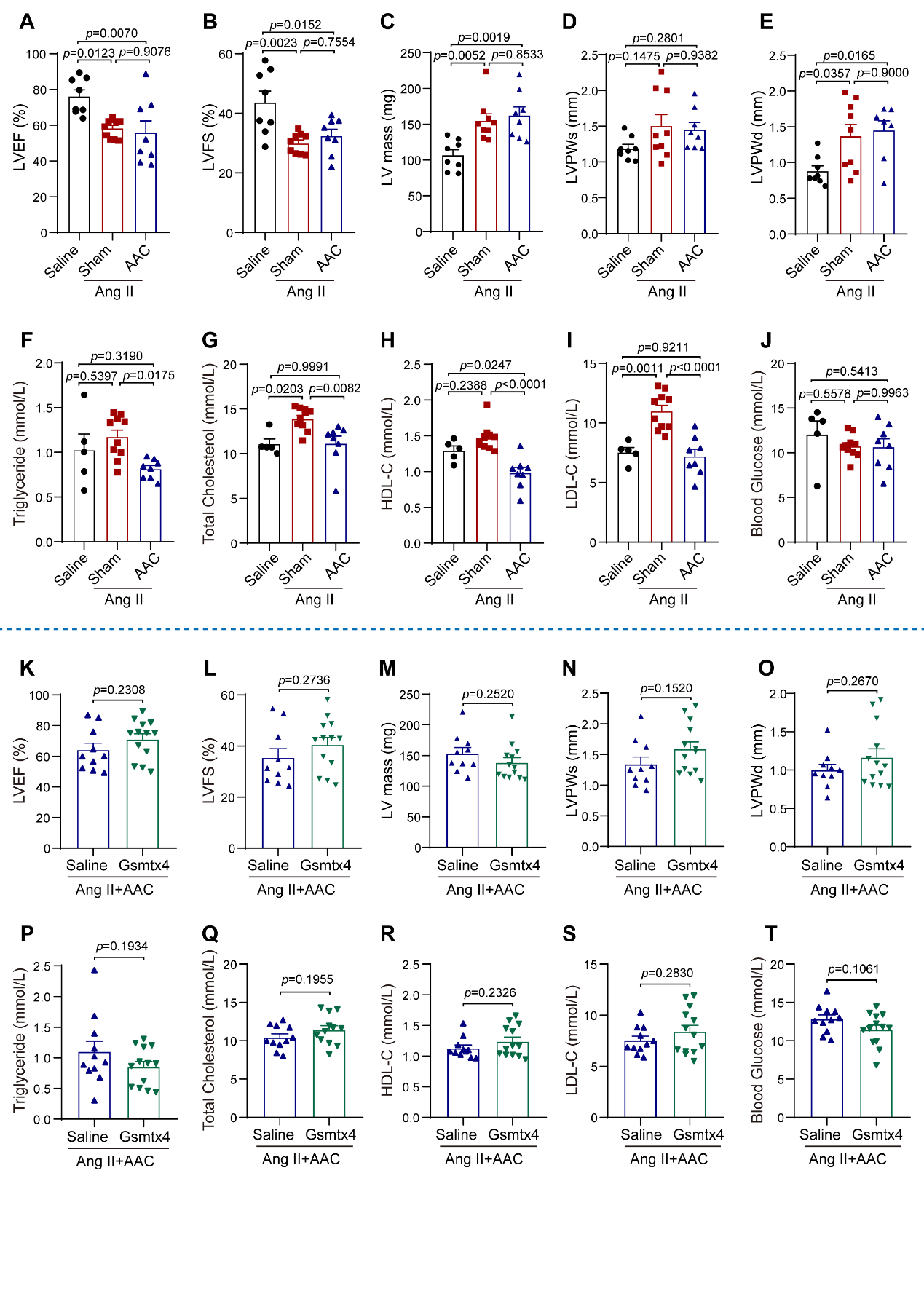


**Figure S2. Cardiac function and plasma lipid profiles from ApoE^−/−^ mice treated with saline, Ang II+sham, and Ang II+AAC.** **(A)** Left ventricular ejection fractions (LVEF) levels. Data are mean±SEM. One-way ANOVA with Dunnett's post hoc analysis (Saline group, n=8; Ang II+sham group, n=10; Ang II+AAC group, n=8). **(B)** Left ventricular fractional shortening (LVFS) levels. Data are mean±SEM. One-way ANOVA with Dunnett's post hoc analysis (Saline group, n=8; Ang II+sham group, n=9; Ang II+AAC group, n=8). **(C)** Left ventricular mass (LV mass) levels. Data are mean±SEM. One-way ANOVA with Dunnett's post hoc analysis (Saline group, n=8; Ang II+sham group, n=9; Ang II+AAC group, n=8). **(D)** Systolic left ventricular posterior wall thickness (LVPWs) levels. Data are mean±SEM. One-way ANOVA with Dunnett's post hoc analysis (Saline group, n=8; Ang II+sham group, n=9; Ang II+AAC group, n=8). **(E)** Diastolic left ventricular posterior wall thickness (LVPWd) levels. Data are mean±SEM. One-way ANOVA with Dunnett's post hoc analysis (Saline group, n=8; Ang II+sham group, n=9; Ang II+AAC group, n=8). **(F)** Triglyceride levels. **(G)** Total cholesterol levels. **(H)** High-density lipoprotein cholesterol (HDL-C) levels. **(I)** Low-density lipoprotein cholesterol (LDL-C) levels. **(J)** Blood glucose levels. Data are mean±SEM. One-way ANOVA with Dunnett's post hoc analysis in F through J (Saline group, n=5; Ang II+sham group, n=10; Ang II+AAC group, n=8). **(K)** LVEF levels. **(L)** LVFS levels. **(M)** LV mass levels. **(N)** LVPWs levels. **(O)** LVPWd levels. Data are mean±SEM. Unpaired Student’s t-test in K through O (Saline group, n=10; GsMTx4 group, n=13). **(P)** Triglyceride levels. **(Q)** Total cholesterol levels. **(I)** HDL-C levels. **(S)** LDL-C levels. **(T)** Blood glucose levels. Data are mean±SEM. Unpaired Student’s t-test in P through T (Saline group, n=11; GsMTx4 group, n=13).

**Figure S3**


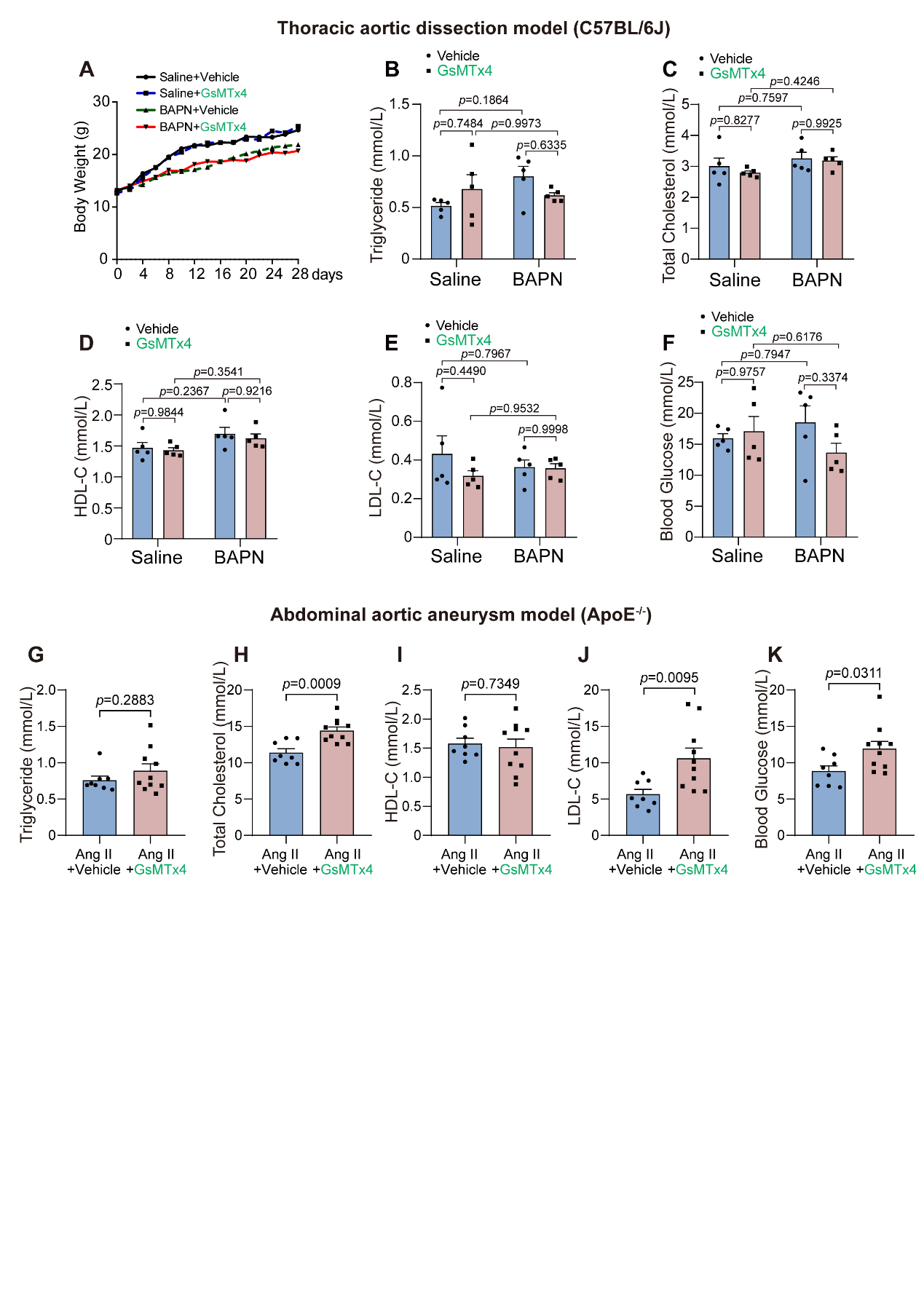


**Figure S3. Body weight and plasma lipid profiles from BAPN- and Ang II-induced AAD mice treated with GsMTx4.** **(A)** Body weight in indicated groups (Saline+Vehicle, n=5; Saline+GsMTx4, n=5; BAPN+Vehicle, n=11; and BAPN+GsMTx4, n=10). **(B)** Triglyceride levels. **(C)** Total cholesterol levels. **(D)** HDL-C levels. **(E)** LDL-C levels. **(F)** Blood glucose levels. Data are mean±SEM. Two-way ANOVA with Dunnett's post hoc analysis in B through F (Saline+Vehicle, n=5; Saline+GsMTx4, n=5; BAPN+Vehicle, n=5; and BAPN+GsMTx4, n=5). **(G)** Triglyceride levels. **(H)** Total cholesterol levels. **(I)** HDL-C levels. **(J)** LDL-C levels. **(K)** Blood glucose levels. Data are mean±SEM. Unpaired Student’s t-test in G through K (Ang II+Vehicle, n=8; Ang II+ GsMTx4, n=10).

**Figure S4**


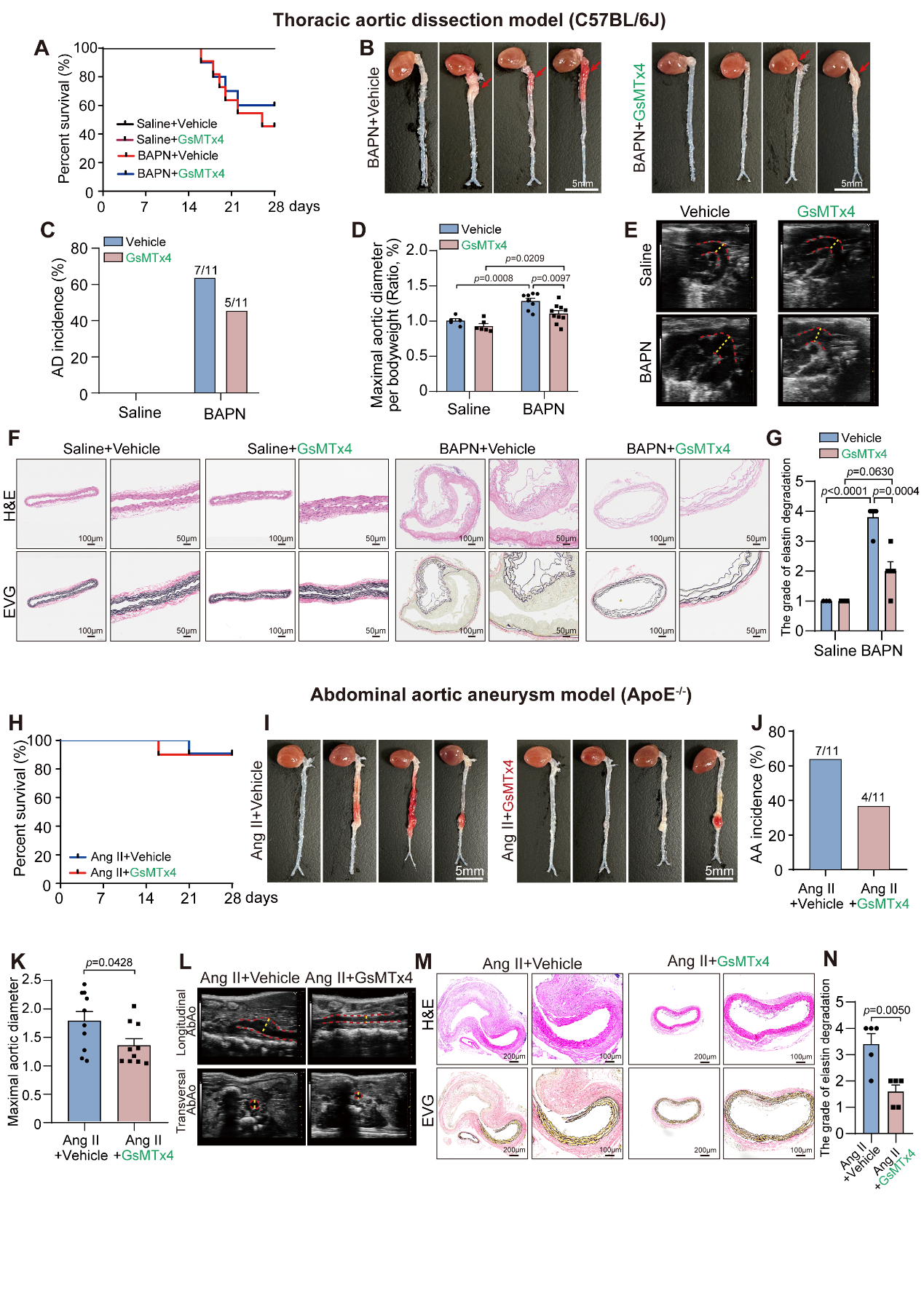


**Figure S4. Inhibition of Piezo1 ameliorates BAPN- and Ang II-induced AAD formation *in vivo.* (A)** The 3-week-old male C57BL/6J mice were constructed BAPN-induced TAD model and were infused with DMSO or the Piezo1 inhibitor GsMTx4 (10 mg/kg) every other day. Survival curves in indicated groups (Saline+Vehicle, n=5; Saline+GsMTx4, n=5; BAPN+Vehicle, n=11; and BAPN+GsMTx4, n=11). **(B)** Representative macroscopic images between Vehicle and GsMTx4 mice 28 days post-BAPN treatment. Scale bar, 5 mm. **(C)** AAA incidence in four male animal groups 28 days post-BAPN treatment (Saline+Vehicle, n=5; Saline+GsMTx4, n=5; BAPN+Vehicle, n=11; and BAPN+GsMTx4, n =11). **(D)** Maximal aortic diameter per bodyweight ratio in four male animal groups 28 days post-BAPN treatment. Data are presented as mean±SEM. Two-way ANOVA with Dunnett's post hoc analysis (Saline+Vehicle, n=5; Saline+GsMTx4, n=5; BAPN+Vehicle, n=8; and BAPN+GsMTx4, n =10). **(E)** Representative ascending aortic ultrasound images in 4 animal groups after 4 weeks following BAPN infusion. **(F)** Representative H&E and EVG staining of mouse thoracic aorta in indicated groups 28 days post-BAPN treatment. Low-magnification images in F show the entire vascular wall at the site of analysis, scale bar, 100 μm. Scale bar of high-magnification images in F, 50 μm. **(G)** Grade of elastin degradation in the aortic wall. Data are presented as mean±SEM. Two-way ANOVA with Dunnett's post hoc analysis (Saline+Vehicle, n=3; Saline+GsMTx4, n=3; BAPN+Vehicle, n=5; and BAPN+GsMTx4, n=5). **(H)** The 8-week-old male ApoE^−/−^ mice were constructed Ang II-induced AAA model and were infused with Vehicle or the Piezo1 inhibitor GsMTx4 (10 mg/kg) every other day. Survival curves in indicated groups (Ang II+Vehicle, n=11; Ang II+GsMTx4, n=10). **(I)** Representative macroscopic images between Vehicle and GsMTx4 mice 28 days post-Ang II treatment. Scale bar, 5 mm. **(J)** AAA incidence in two groups 28 days post-Ang II treatment (Ang II+Vehicle, n=11; Ang II+ GsMTx4, n=11). **(K)** Maximal aortic diameter in two groups 28 days post-Ang II treatment. Data are presented as mean±SEM. Unpaired Student’s t-test (Ang II+Vehicle, n=10; Ang II+ GsMTx4, n=10). **(L)** Representative abdominal aorta ultrasound images in two animal groups at 4 weeks following Ang II infusion. **(M)** Representative H&E and EVG staining of mouse abdominal aorta in indicated groups 28 days post-Ang II treatment. Low-magnification images in M show the entire vascular wall at the site of analysis, scale bar, 200 μm. Scale bar of high-magnification images in M, 100 μm. **(N)** Grade of elastin degradation in the aortic wall. Data are presented as mean±SEM. Unpaired Student’s t-test (Ang II+Vehicle, n=5; Ang II+ GsMTx4, n=5).

**Figure S5**


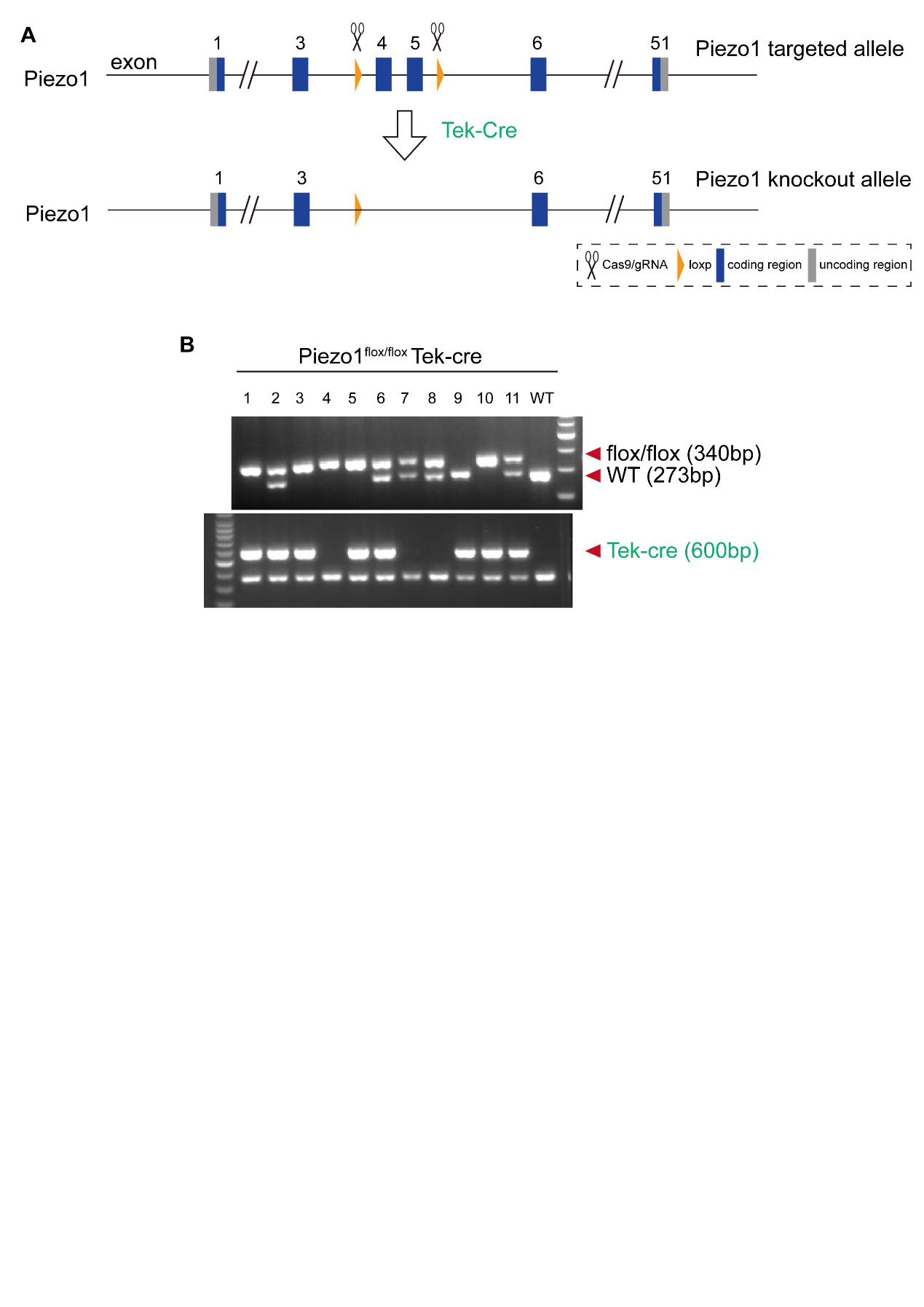


**Figure S5.** **Construction of** **Piezo1^ΔEC^ mouse and genotype identification. (A)** Overview of EC-specific Piezo1 knockout (Piezo1^ΔEC^) mouse construction. **(B)** Mouse genotype identification. PCR analysis of Piezo1^flox^ and Tek-Cre mice. The black font refers to the loxp band (Mutant band is in 340 bp and WT band is in 273 bp). The green font refers to Cre band (Cre band is in 600 bp).

**Figure S6**


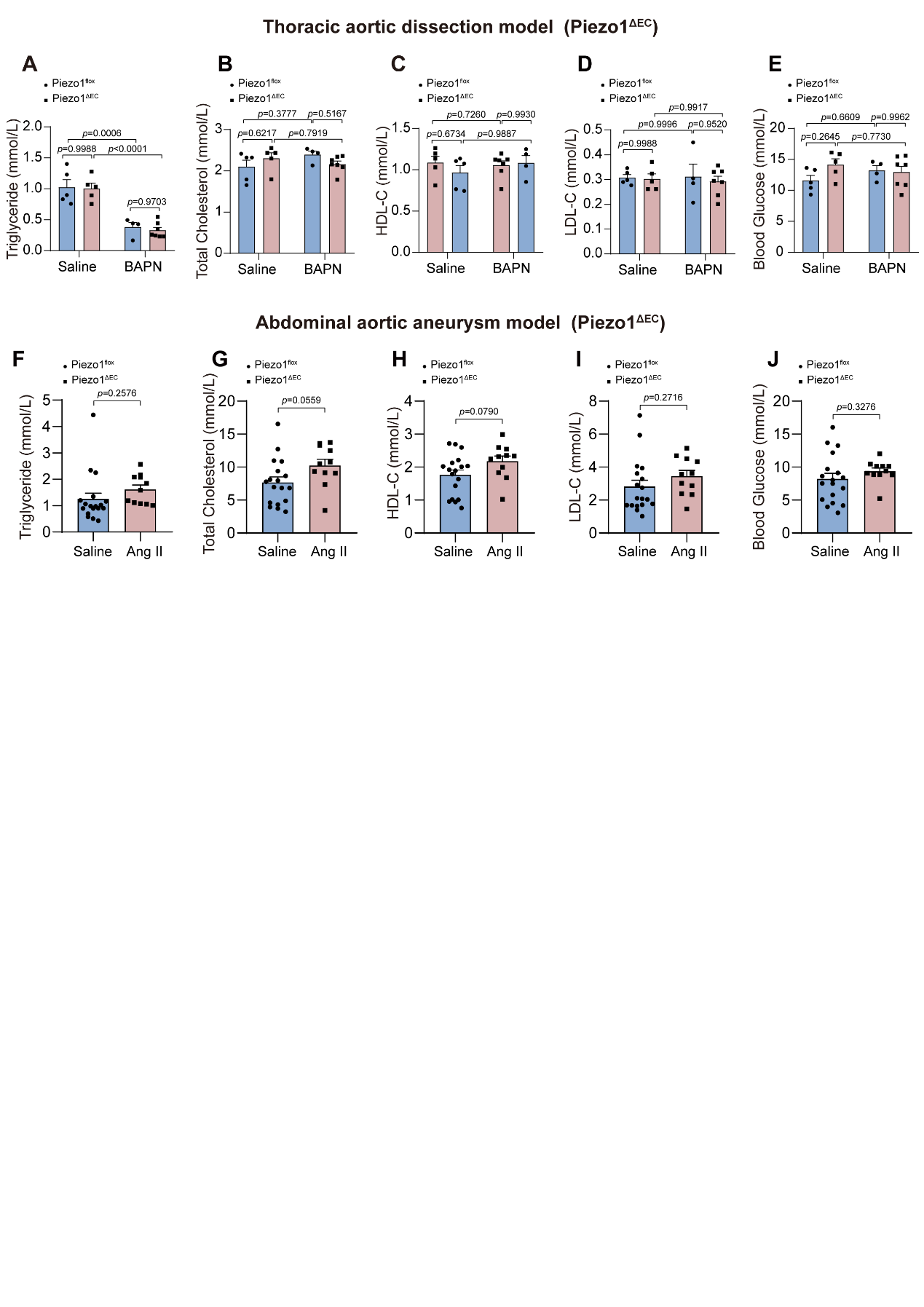


**Figure S6.** **Effect of ECs-specific Piezo1 knockout on BAPN- and Ang II-induced changes in plasma lipid profiles. (A)** Triglyceride levels. **(B)** Total cholesterol levels. **(C)** HDL-C levels. **(D)** LDL-C levels. **(E)** Blood glucose levels. Data are mean±SEM. Two-way ANOVA with Dunnett's post hoc analysis in A through E (Saline+Piezo1^ﬂox^, n=5; Saline+ Piezo1^ΔEC^, n=5; BAPN+Piezo1^ﬂox^, n=4; and BAPN+Piezo1^ΔEC^, n=7). **(F)** Triglyceride levels. **(G)** Total cholesterol levels. **(H)** HDL-C levels. **(I)** LDL-C levels. **(J)** Blood glucose levels. Data are mean±SEM. Unpaired Student’s t-test in F through J (Ang II+ Piezo1^ﬂox^, n=18; Ang II+ Piezo1^ΔEC^, n=11).

**Figure S7**


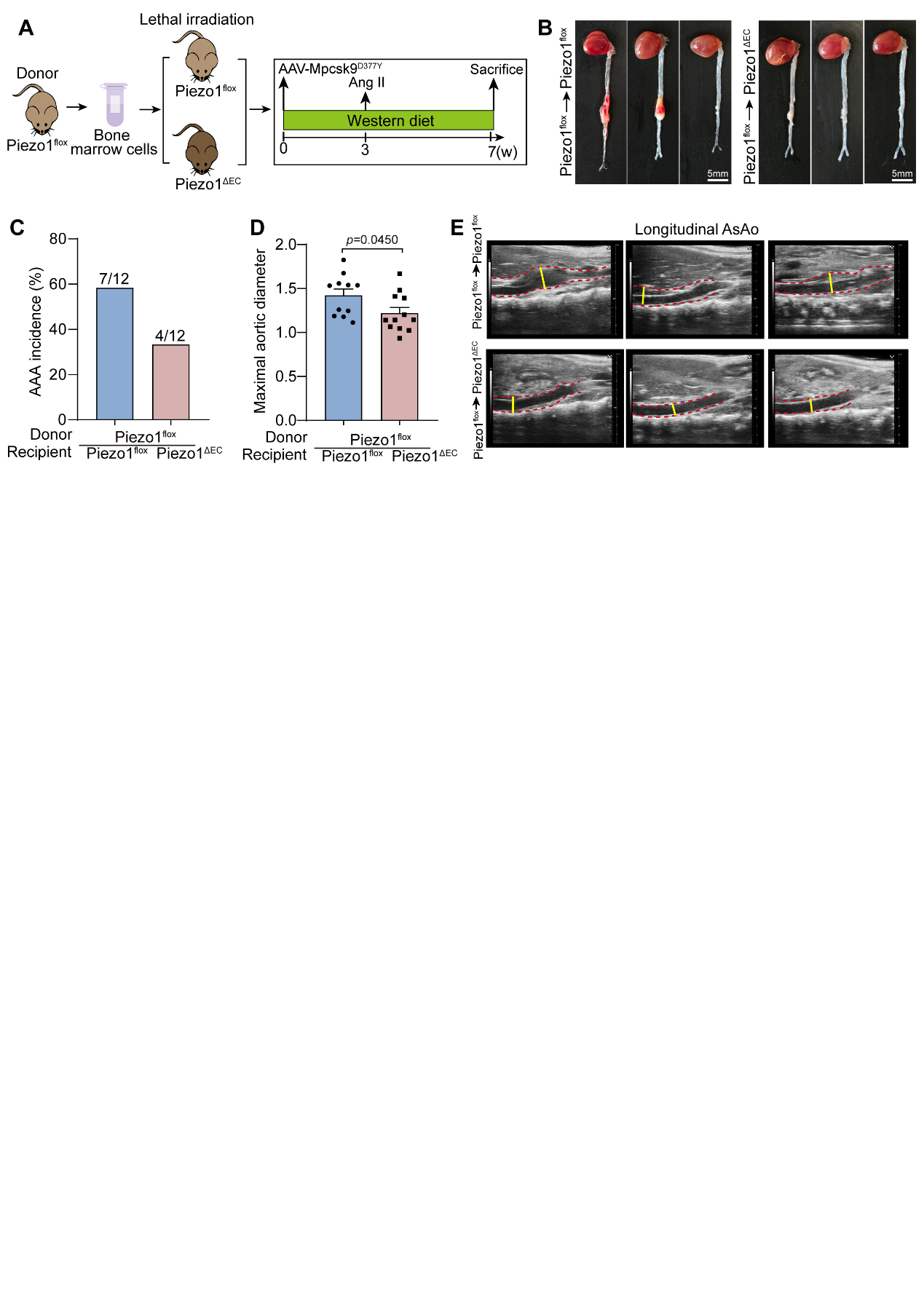


**Figure S7. Endothelial Piezo1 deficiency alone markedly attenuated AAA development. (A)** Experimental strategy of BMT experiments. **(B)** The 8-week-old male Piezo1^ﬂox^ and Piezo1^ΔEC^ mice (both transplanted with Piezo1^ﬂox^ bone marrow) were injected with AAV8-PCSK9^D377Y^ prior to Ang II infusion. Representative macroscopic images of the abdominal aorta from Piezo1^ﬂox^ and Piezo1^ΔEC^ mice transplanted Piezo1^ﬂox^ BM 28 days post-Ang II treatment. Scale bar, 5 mm. **(C)** AAA incidence in two groups 28 days post-Ang II treatment (Piezo1^ﬂox^ mice transplanted Piezo1^ﬂox^ BM, n=12; Piezo1^ΔEC^ mice transplanted Piezo1^ﬂox^ BM, n=12). **(D)** Maximal aortic diameter in two male animal groups 28 days post-Ang II treatment. Data are presented as mean±SEM. Unpaired Student’s t-test (Piezo1^ﬂox^ mice transplanted Piezo1^ﬂox^ BM, n=11; Piezo1^ΔEC^ mice transplanted Piezo1^ﬂox^ BM, n=12). **(E)** Representative abdominal aorta ultrasound images in two male animal groups at 4 weeks following Ang II infusion.

**Figure S8**


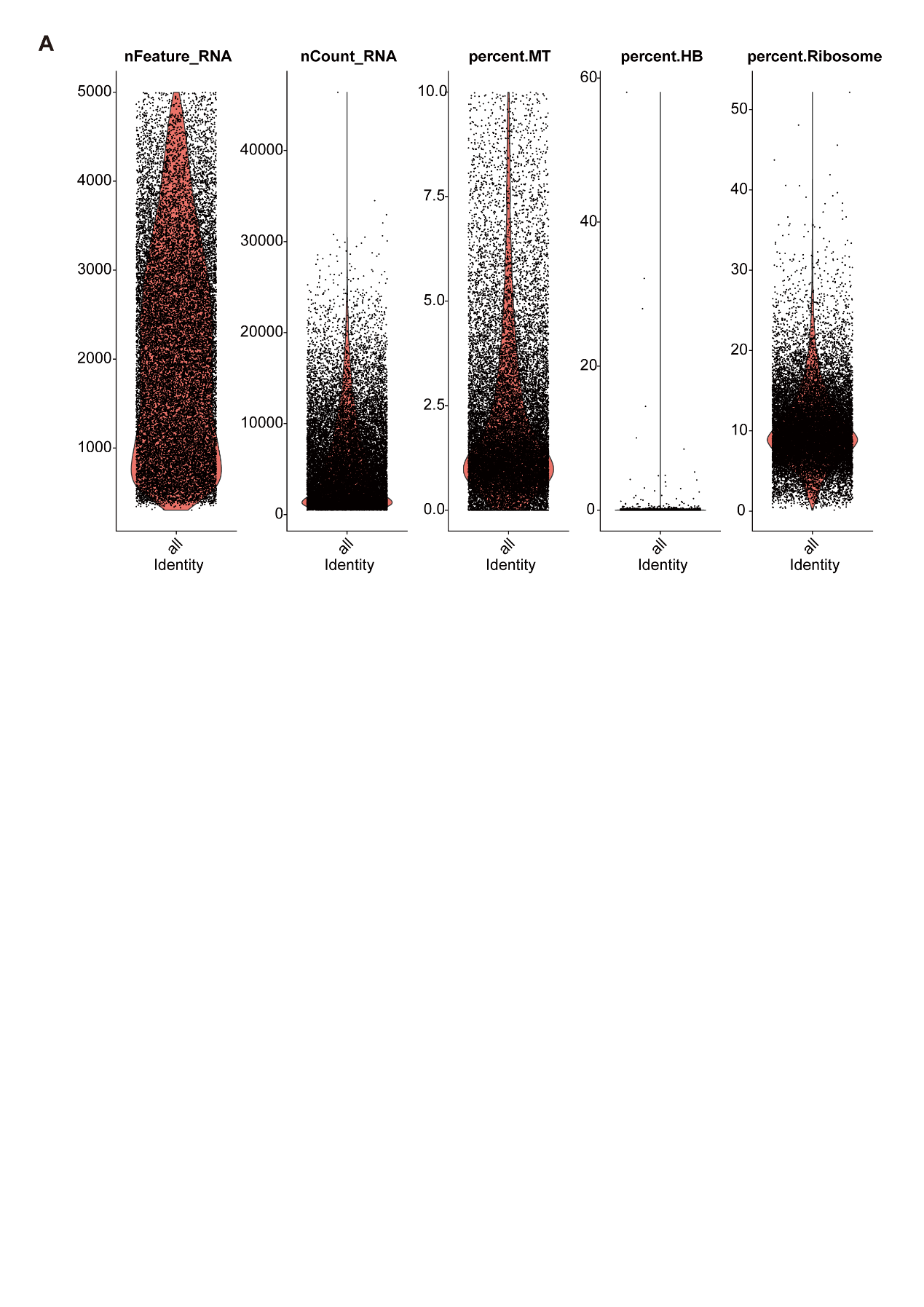


**Figure S8.** **Quality Control (QC) plots.** **(A)** The distribution of the number of genes, number of counts and percentage of mitochondrial fraction in two samples were showed by violin plots. Cells were filtered out if more than 5,000 genes, less than 300 genes were detected, the percentage of mitochondrial gene counts higher than 10%.

**Figure S9**


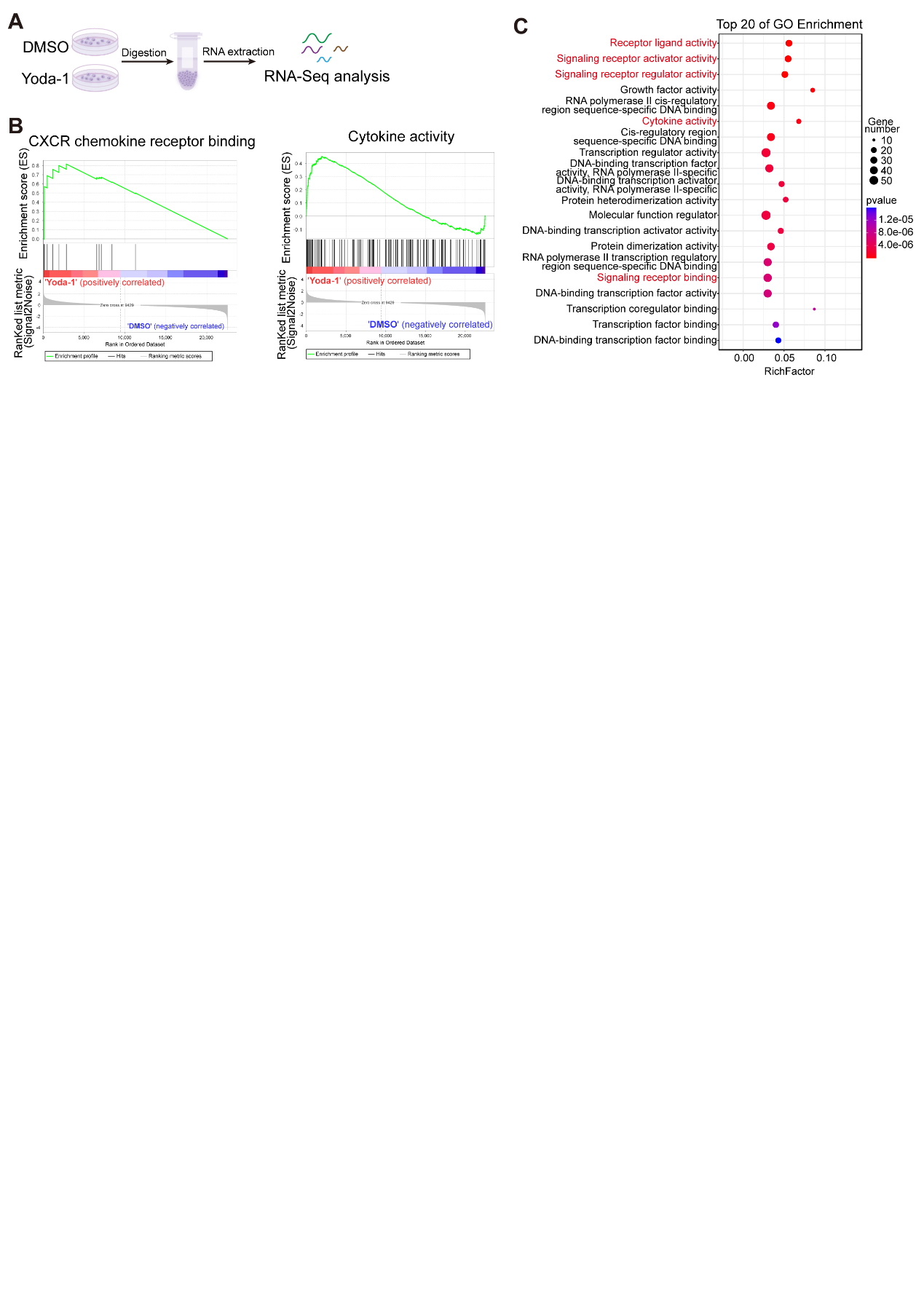


**Figure S9.** **Piezo1 activation promotes endothelial inflammation and leukocyte recruitment. (A)** Flowchart of transcriptomic analysis (mRNA sequencing) in human aortic endothelial cells (HAECs). **(B)** Gene Set Enrichment Analysis (GSEA) showing the CCXCR chemokine receptor binding pathway and cytokine pathway associated with the upregulated differentially expressed genes in HAECs after Yoda-1 stimulation. **(C)** Bubble plot showing the top 20 enriched GO pathways associated with the upregulated differentially expressed genes in HAECs after Yoda-1 stimulation.

**Figure S10**


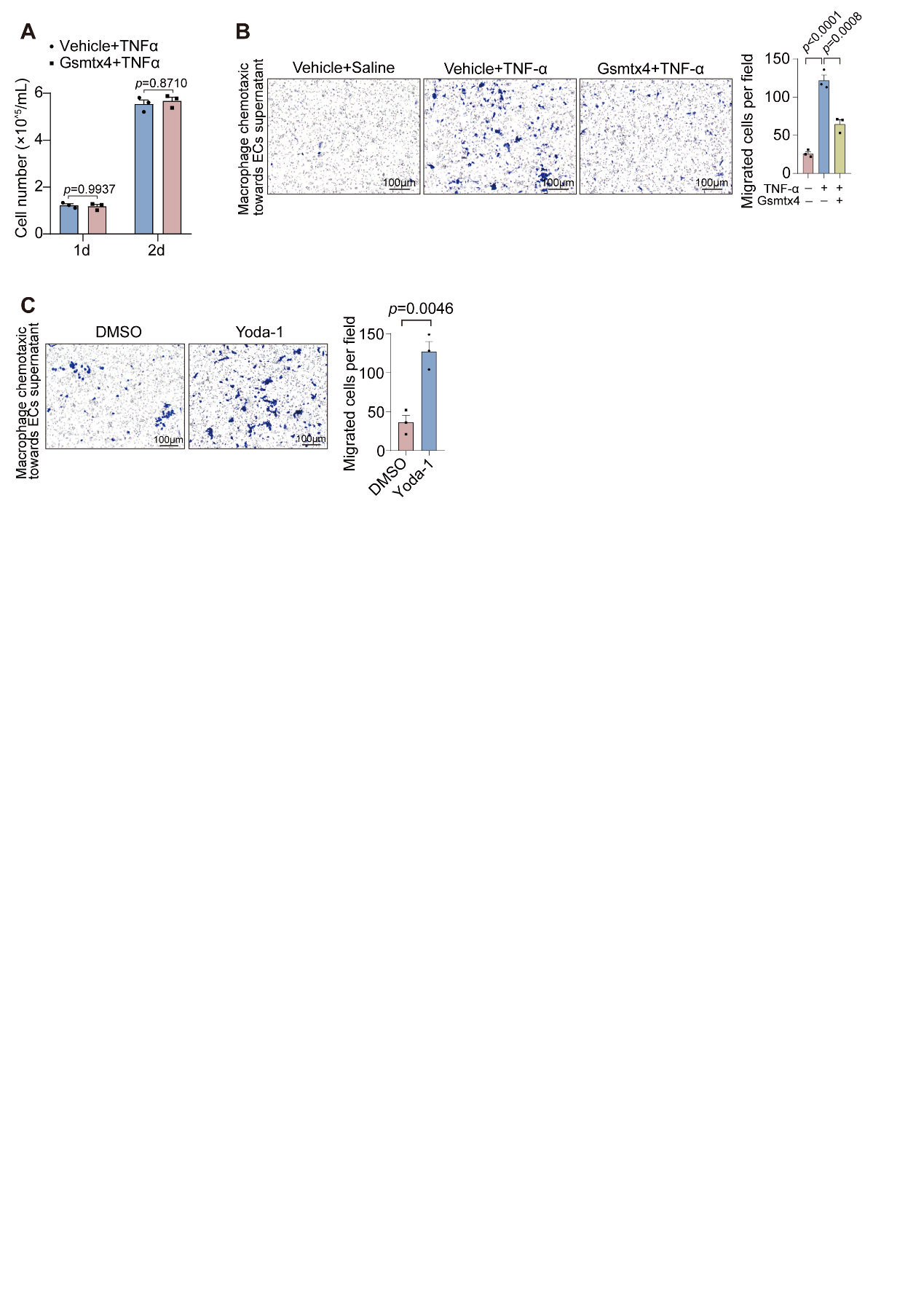


**Figure S10. Piezo1 promotes macrophage migration toward ECs *in vitro*. (A)** Total number of monocytes treated with Vehicle+TNF-α and Gsmtx4+TNF-α at the indicated times. Data are presented as mean±SEM. Unpaired Student’s t-test (n=3 per group). **(B)** Representative images and quantitative analysis of Transwell assays of macrophages. Cells migrating to the bottom were stained with crystal violet. Scale bars, 100 μm. Data are presented as mean±SEM. One-way ANOVA with Dunnett's post hoc analysis (n=3 per group). **(C)** Representative images and quantitative analysis of Transwell assays of macrophages. Cells migrating to the bottom were stained with crystal violet. Scale bars, 100 μm. Data are presented as mean±SEM. Unpaired Student’s t-test (n=3 per group).

**Figure S11**


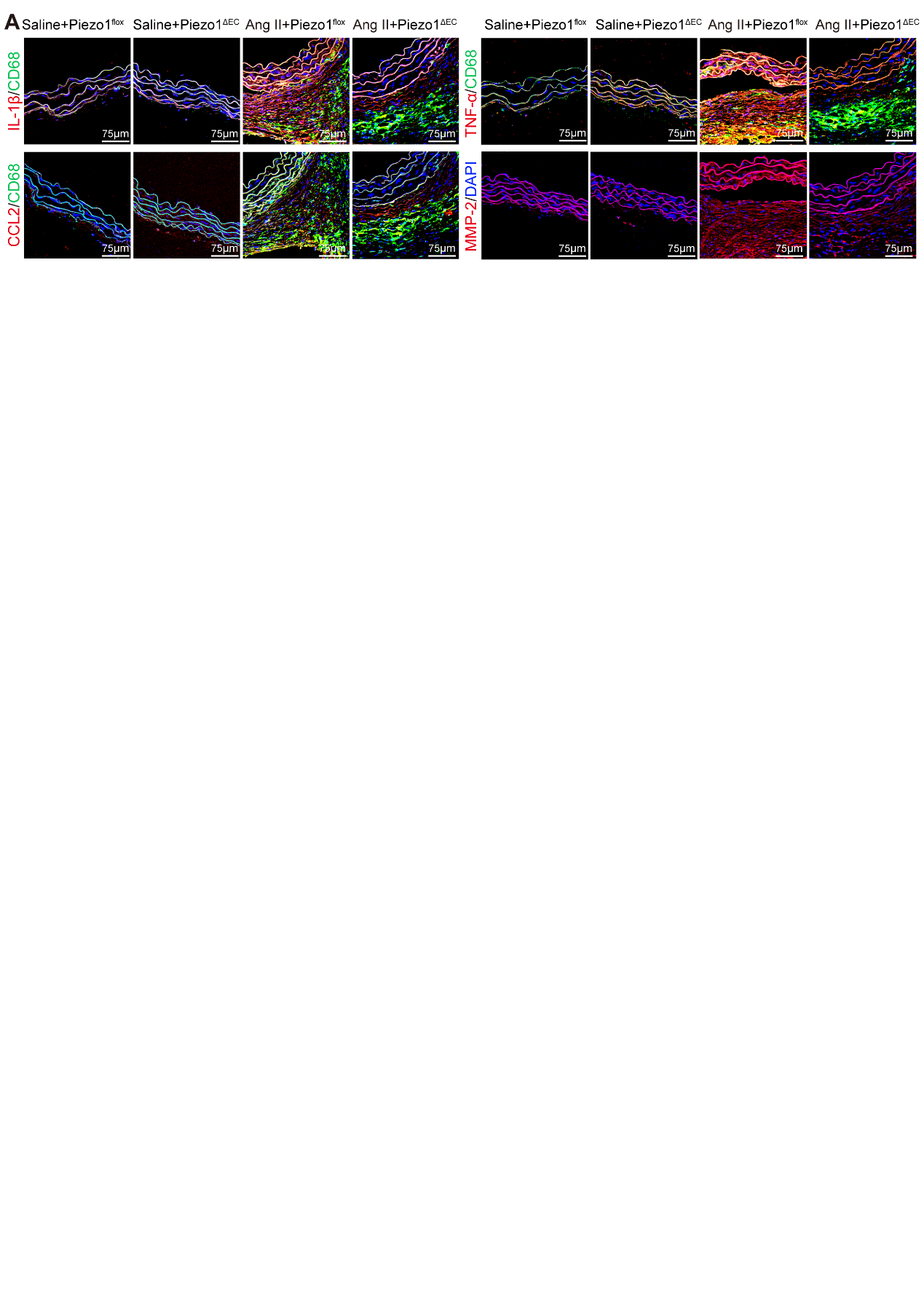


**Figure S11. EC-specific Piezo1 deficiency represses Ang II-induced inflammatory response. (A)** Representative images showing immunostaining for CD68 (a marker of macrophage, green) and IL-1β, CCL2, TNF-α, and MMP-2 in AAA lesions of Piezo1^ﬂox^ and Piezo1^ΔEC^ mice treated with Ang II for four weeks (n=4 per group). DAPI (blue) was used for nucleic acid labeling. Scale bar=75 μm.

**Figure S12**


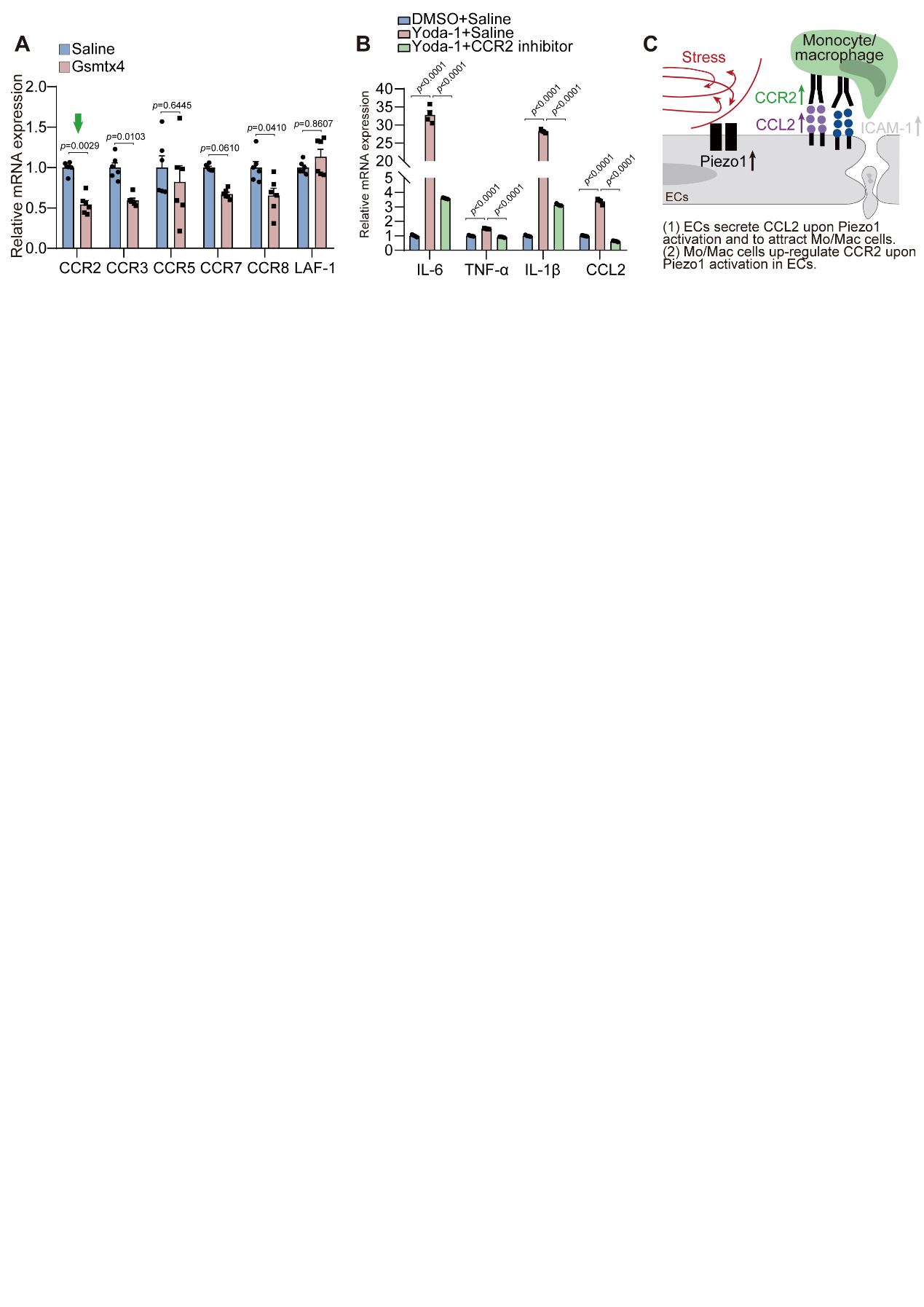


**Figure S12. Piezo1 facilitates CCR2 in macrophages and blocking CCR2 with an inhibitor suppresses inflammation. (A)** Cultured macrophages were stimulated with vehicle and Gsmtx4. Cells were harvested and C-C motif chemokine receptors mRNA expression were detected by RT-qPCR. Data are presented as mean±SEM. Unpaired Student’s t-test (n=6 per group). **(B)** Cultured macrophages were stimulated with DMSO+Saline, Yoda-1+Saline, and Yoda-1+CCR2 inhibitor. Cells were harvested and IL-6, TNF-α, IL-1β, and CCL2 mRNA expression were detected by RT-qPCR. Data are presented as mean±SEM. One-way ANOVA with Dunnett's post hoc analysis (n=4 per group). **(C)** Schematic representation showing how fluid shear stress activates Piezo1 in ECs to induce facilitate CCL2-CCR2 chemoattraction and subsequently the monocyte/macrophage trans-endothelial migration.

**Figure S13**
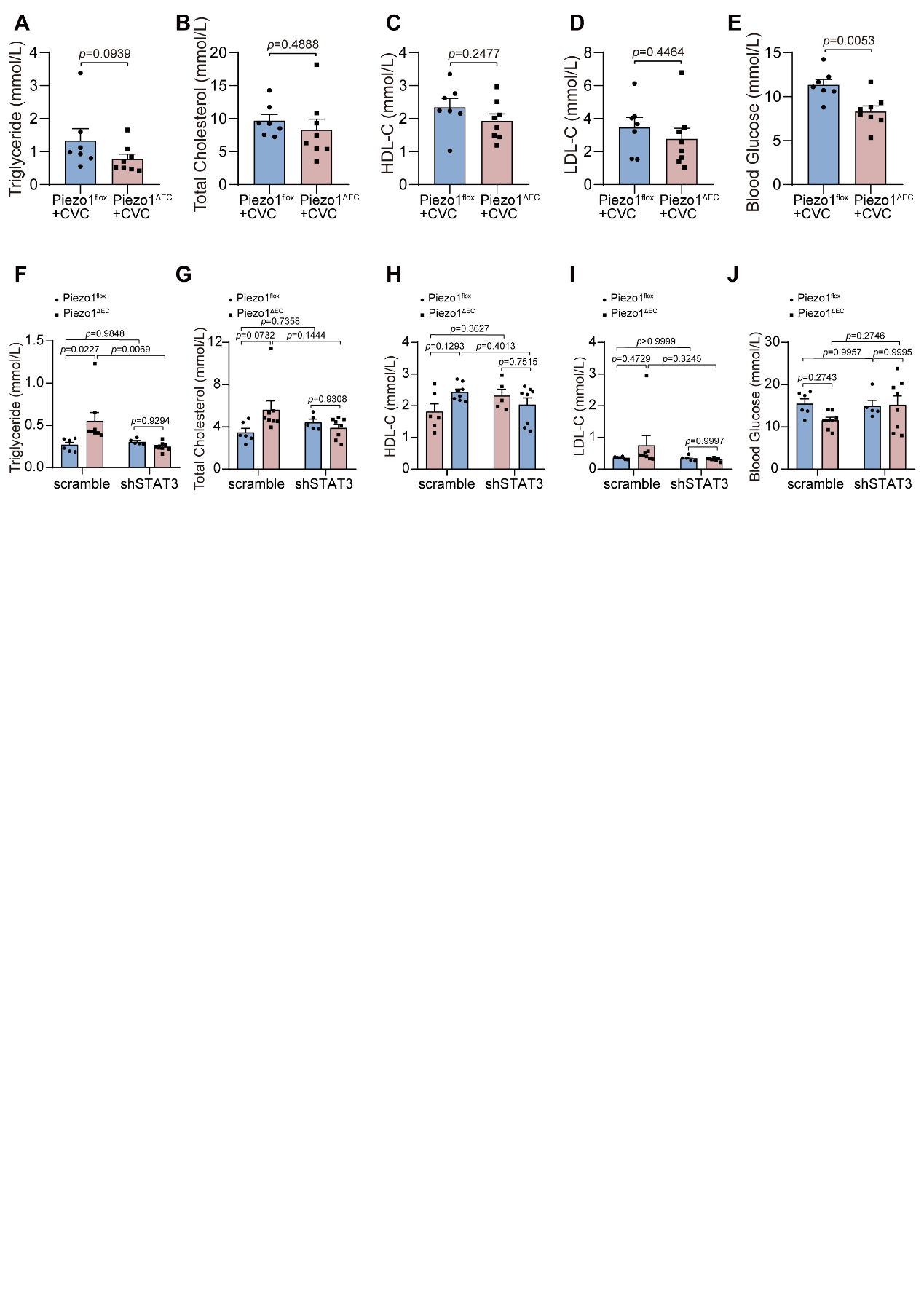


**Figure S13. Plasma lipid profiles from** **Piezo1^ﬂox^ and Piezo1****^ΔEC^ treated with CVC or shSTAT3. (A)** Triglyceride levels. **(B)** Total cholesterol levels. **(C)** HDL-C levels. **(D)** LDL-C levels. **(E)** Blood glucose levels. Data are mean±SEM. Unpaired Student’s t-test in A through E (Piezo1^ﬂox^+CVC, n=7; Piezo1^ΔEC^+CVC, n=8). **(F)** Triglyceride levels. **(G)** Total cholesterol levels. **(H)** HDL-C levels. **(I)** LDL-C levels. **(J)** Blood glucose levels. Data are mean±SEM. Two-way ANOVA with Dunnett's post hoc analysis in F through J (Piezo1^ﬂox^+AAV-Scramble, n=6; Piezo1^ΔEC^+AAV-Scramble, n=8; Piezo1^ﬂox^+AAV-shSTAT3, n=5; Piezo1^ΔEC^+ AAV-shSTAT3, n=8).

**Figure S14**


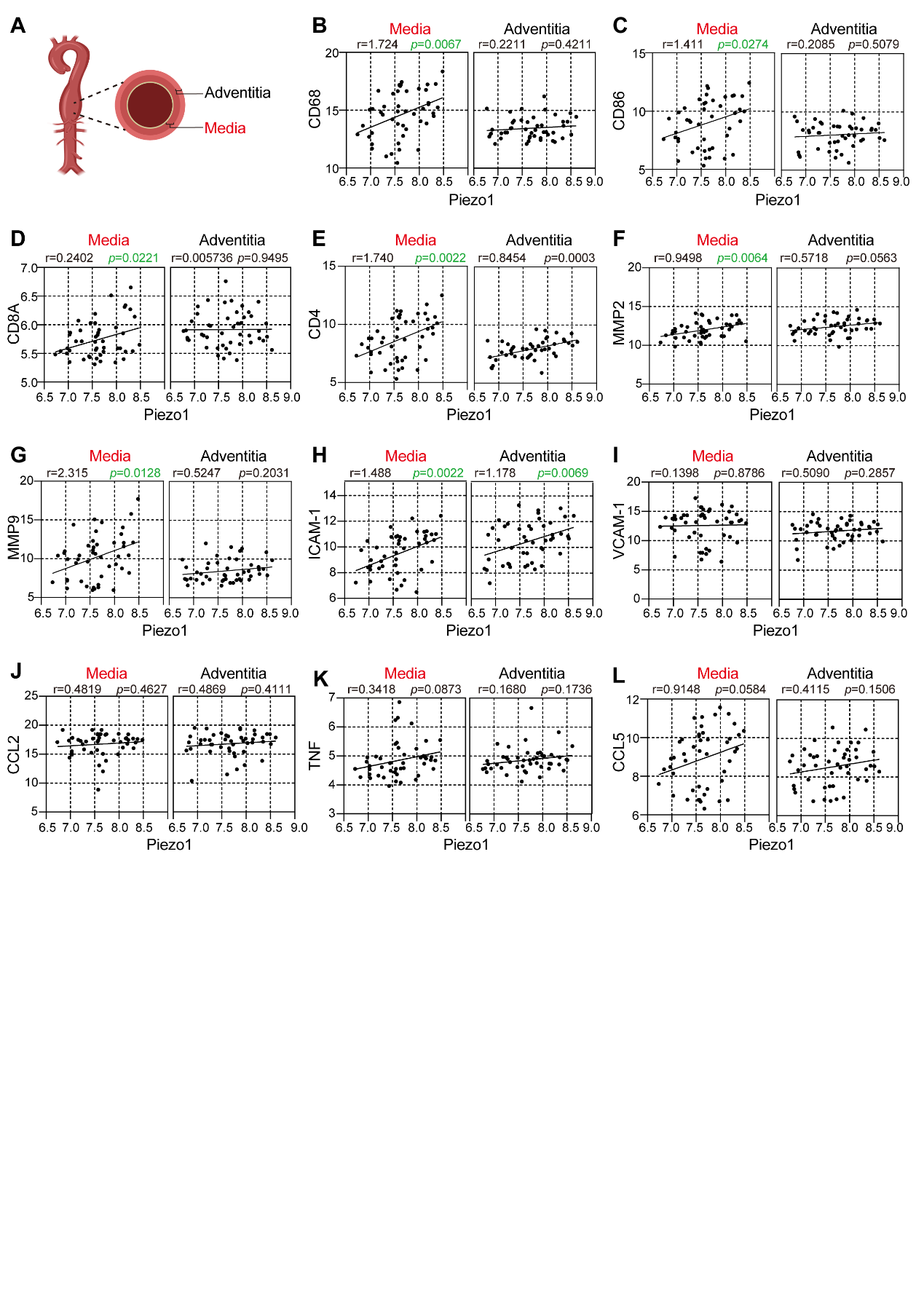


**Figure S14.** **Piezo1 positively correlates with inflammation in patients with AAA. (A)** Schematic depicting anatomical view of the media and adventitia tissue in the aorta. **(B-L)** The correlations between Piezo1 mRNA expression level and CD68 **(B)**, CD86 **(C)**, CD8A **(D)**, CD4 **(E)**, MMP2 **(F)**, MMP9 **(G)**, ICAM1 **(H)**, VCAM1**(I)**, CCL2 **(J)**, TNF **(K)**, and CCL5 **(L)** mRNA expression in medial (left) and adventitial (right) tissues in patients with AAA (n = 34) and control (n = 13) are shown. The correlation between two genes was analyzed using the Pearson correlation, and Pearson correlation coefficient is referred to as r. *p* value and r (microarray analysis from GSE232911) were calculated using GraphPad Prism version 8.0. r and *p* values are labelled in each individual figure. Statistically significant *p* value (<0.05) is highlighted in green.

**Figure S15**


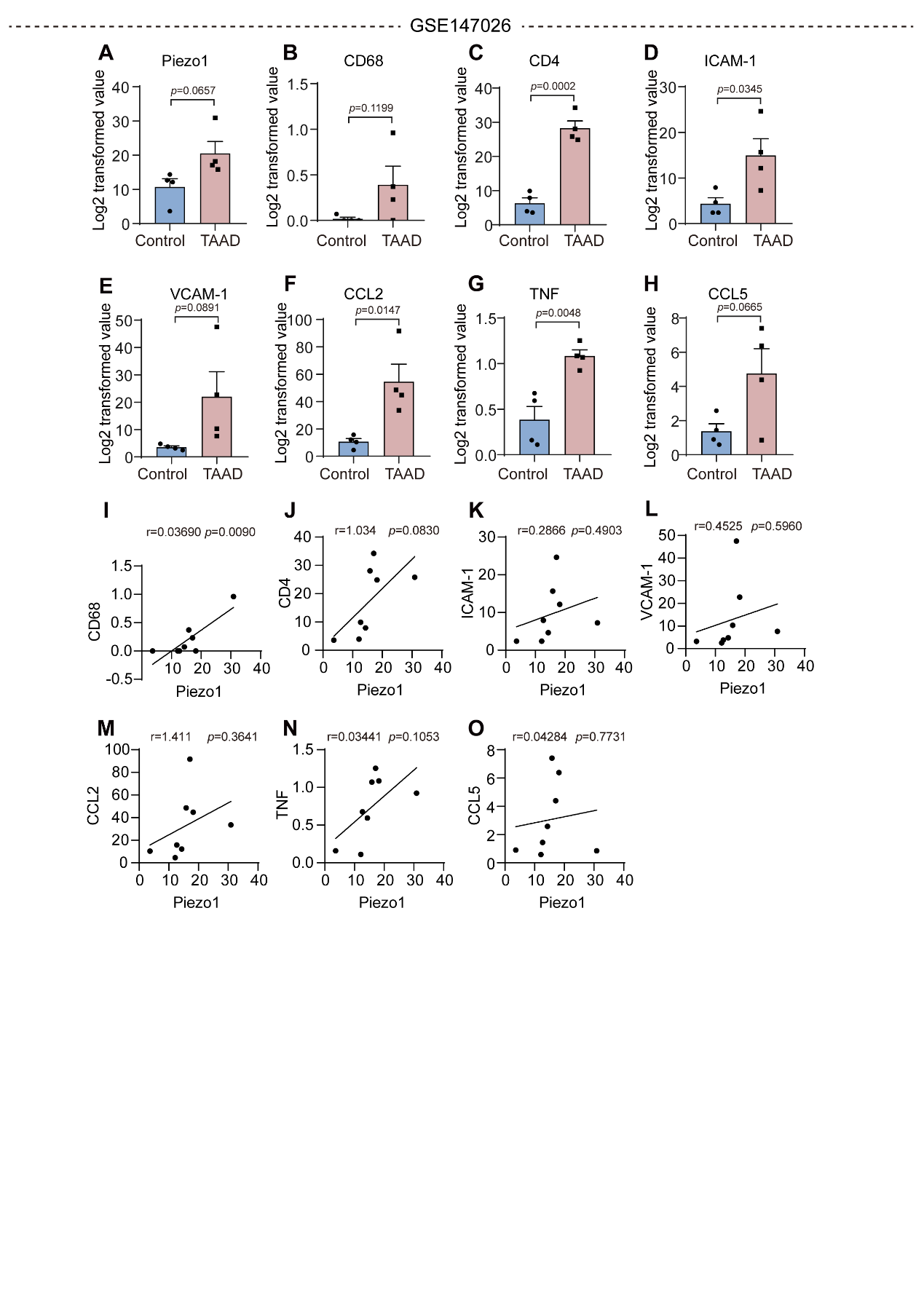


**Figure S15. Piezo1 positively correlates with inflammation in patients with TAD. (A-H)** The Piezo1 mRNA expression level (A) and CD68 (B), CD4 (C), ICAM1 (D), VCAM1(E), CCL2 (F), TNF (G), and CCL5 (H) mRNA expression in medial tissues in patients with TAD and control are shown. Unpaired Student’s t-test in A through H (control, n=4; TAD, n=4). **(I-O)** The correlations between Piezo1 mRNA expression level and CD68 (I), CD4 (J), ICAM1 (K), VCAM1(L), CCL2 (M), TNF (N), and CCL5 (O) mRNA expression in medial tissues in patients with TAD (n = 4) and control (n = 4) are shown. The correlation between two genes was analyzed using the Pearson correlation, and Pearson correlation coefficient is referred to as r. *p* value and r (microarray analysis from GSE147026) were calculated using GraphPad Prism version 8.0. r and *p* values are labelled in each individual figure.

**Supplementary methods**

**Human tissues**

This study was approved by the Medical Institutional Ethics Committee of Qilu Hospital, Shandong University, China (KYLL-202410-038). Legal representatives of all participants or organ donors provide written informed consent prior to registration.

Human thoracic aortic control and abdominal aortic control samples were collected from nine organ donors (males). All donors were deceased as a result of acute trauma or cerebral hemorrhage. The donors ranged in age from 37 to 65 years, with a median age of 51.89 years. Human thoracic aortic aneurysm (TAA), thoracic aortic dissection (TAD), and abdominal aortic aneurysm (AAA) samples were obtained from nine AAD patients (nine males and three females) who underwent open surgical repair. Patients with AAD ranged in age from 47 to 79 years, with a median age of 58.58 years. These samples were used for immunofluorescence, immunohistochemical, and western blot validation. The aortas of both controls and AAD patients were collected using the same technical conditions and were dissected carefully to reduce the likelihood of cell damage from surgery. Information regarding these tissue samples is provided in Table S1.

**Genetic Mouse Models**

The Piezo1^flox^ (Tek-Cre^−/−^; Piezo1^flox/flox^) mice were hybridized with Tek-Cre mice to induce endothelial cell-specific Piezo1 knockout mice (Piezo1^ΔEC^). This genetic mouse model was generated on the C57BL/6J (WT) background at the Shanghai Model Organisms Center.

The sample size for each experiment is described in the figure legends section. All mouse manipulations were performed following the recommendations in the Guide for the Care and Use of Laboratory Animals published by the US National Institutes of Health (NIH publication no. 85-23 revised1996) and approved by the Ethics Committee and the Scientific Investigation Board of Qilu Hospital, Shandong University, China (KYLL-202410-038).

**AAD Mouse Models**

Because female mice were naturally resistant to Ang II treatment and presented significantly lower AAA incidence, only male mice were used in this study.

AAA describes a weakening and dilatation of the abdominal aorta, most commonly affecting the infrarenal part. The main method used to induce AAA in mice is subcutaneous infusion of Ang II on different gene backgrounds[^34^](#_ENREF_34). TAD occurs when a tear forms within the aortic wall and causes blood to flow between the laminar layers of the media, thereby separating them and creating a false lumen with a severely weakened outer aortic wall. The main method used to induce TAD in mice is feeding them a normal diet and administering freshly prepared BAPN solution dissolved in the drinking water (0.25%) for 4 weeks on C57BL/J background[^43^](#_ENREF_43). Since none of the existing animal models can completely mimic all aspects of human AAD, leveraging multiple models offers a robust approach to elucidate the role of Piezo1 in AAD.

The PCSK9/Ang II-induced murine AAA model was performed as previously described[^44^](#_ENREF_44). In brief, 8-10 weeks male mice were administered via the lateral tail intravenous (IV) injections of 2×10^11^ genomic copies of adeno-associated virus (AAV, serotype 8) carrying a gain-of-function mutation of the mouse Pcsk9 (AAV.Mpcsk9^D377Y^) and food was changed to a western diet (HCD, 17.3% protein, 21.2% fat, 48.5% carbohydrate, 0.2% cholesterol by mass, and 42% calories from fat; TD.88137, Envigo) immediately after injection to induce hyperlipidemia. Three weeks after AAVs injection, Osmotic pumps (Alzet, model 2004) containing Ang II (1000 ng/kg/min, MCE, HY-13948) were implanted and the western diet was continued for 4 weeks to induce AAA formation.
 The Ang II-induced murine AAA model on ApoE^-/-^ background was generated using Osmotic pumps (Alzet, model 2004) containing either Ang II (1000 ng/kg/min, MCE, HY-13948) or saline for infusion for 4 weeks in ApoE^-/-^ male mice.

In the BAPN-induced murine TAD model, 3-4 weeks old male mice were given water containing 0.25% BAPN (0.8g/kg/day, Sigma, A3134) for 4 weeks.

Anesthesia in mice was conducted by 3% isoflurane induction and 1.5% isoflurane maintenance. Finally, mice were euthanized by intravenous injection of a lethal dose of pentobarbital sodium (100 mg/kg). Abdominal aortas harvested were snap-frozen in liquid nitrogen and then stored at -80 °C pending further processing. We provided a summary table outlining findings across all models for easier comparison (Table S2).

**Construction of abdominal aorta coarctation model**

The AAC model was built by following these steps. The mice fasted 8 hours before surgery and drank water freely. After anesthesia, mice were supine fixed on a constant temperature operating table, and the abdominal operation area was skinned and disinfected. The skin was cut layer by layer at 2-3 cm below the xiphoid process to expose the abdominal cavity and the left kidney. The aorta was bluntly separated above the level of the renal vein, and the 4-0 surgical suture was inserted below the aorta for reserve. A 26G needle was placed parallel to the abdominal aorta closely to the blood vessel, and the needle and aorta were ligated together with the suture. Then lift both stitches and gently pull out the needle and cut the long suture threads. After checking the abdominal cavity for missing items, the abdominal organs were returned to the position of the muscle and skin layer by layer for disinfection.

One week before the construction of the abdominal aneurysm model, the abdominal aortic coarctation model (AAC) was constructed by ligation the abdominal aorta above the branch of the renal artery in mice using a 26G safety needle (outer diameter 0.46 mm, inner diameter 0.25 mm), and the aneurysm model was constructed one week after the mice had recovered. The following 3 groups were established: Saline, Ang II+sham, AAC+Ang II. Piezo1 inhibitor GsMTx4 (10 mg/kg, Selleck, p1205) was simultaneously administered every other day, and the following two groups were set up: AAC+Ang II+saline, AAC+Ang II+GsMTx4.

**Single-cell sequencing of mouse aortic tissues**

1) Single cell suspension preparation: mouse aortic samples were routinely prepared for digestion. Aortic tissues were collected from ApoE^-/-^mice before Angiotensin II (Ang II, 1000 ng/kg/min, MCE, HY-13948) infusion on days 3 and 28 post-infusion, or collected from Piezo1^ﬂox^ and Piezo1^ΔEC^ mice after days 28 Ang II infusion. The preparation of single-cell suspensions from aortic cells followed a standard enzymatic digestion protocol. First, aortic tissues were cut and digested with enzyme solution for 1.5 hours at 37 ℃. The cell suspension was filtered through a 30 μm filter and washed twice with PBS. Cells were then resuspended in PBS containing 0.4% BSA, Trypan blue staining confirmed that the cell viability was greater than 80%. Subsequently, the resuspended cells were subjected to scRNA-seq.

2) Cell screening: The R package Seurat was used for dimensionality reduction, clustering and analysis of single-cell RNA sequencing data. Cells were filtered out if more than 5,000 genes, less than 300 genes were detected, the percentage of mitochondrial gene counts higher than 10%.

3) Single cell clustering: Seurat v.4.1.1 was utilized for cluster analysis. To avoid batch effects between samples and experiments, we used Harmony for an ensemble analysis of samples. Gene expression was visualized using Seurat's dot plots.

4) Cell population definition: FindAllMarkers function in Seurat and Wilcoxon rank-sum test were utilized to identify the marker genes of each cluster. The differential genes in EC population from aortic tissues of ApoE^-/-^ mice following 3-day and 28-day Ang II infusion treatment is available in Excel S1, Supporting Information. The differential genes in EC population from aortic tissues of Piezo1^ﬂox^ and Piezo1^ΔEC^ mice following 28-day Ang II infusion treatment is available in Excel S2, Supporting Information.

**Bulk mRNA sequencing**

Total RNA was extracted with Trizol and assessed with the kaiaoK5500®Spectrophotometer (Kaiao, Beijing, China) and the RNA Nano 6000 Assay Kit of the Bioanalyzer 2100 system (Agilent Technologies, CA, USA). Total RNA samples that met the following requirements were used in subsequent experiments: RNA integrity number (RIN) > 7.0 and a 28S:18S ratio > 1.8. The sequencing quality was assessed with FastQC (Version 0.11.5) and then low-quality data were filtered using NGSQC (v0.4), a quality value of over 50% bases of the read was less than 5, and trimmed the adapter sequence, and removed the reads containing poly-N using inhouse Perl scripts. Sequence libraries were generated and sequenced by CapitalBio Technology (Beijing, China). RNA was extracted from ECs after Yoda-1 and DMSO treatment (n=4 per group), and the differential genes list of ECs is available in Excel S3, Supporting Information.

**Echocardiography**

After Ang II or BAPN infusion, the maximal diameter of the aorta was measured with a high-resolution ultrasound imaging system (Visual Sonics, Vevo3100) in a double-blind way. The ruptured aorta was excluded from the analysis of aortic diameter but included in the analysis of AAD incidence. Mice were anesthetized with 1.2-1.5 Vol. % isoflurane.

**Elastin degradation score**

To quantitate elastin degradation, a previously described grading method was used， Grade 1: no elastin degradation; Grade 2: mild elastin degradation; Grade 3: severe elastin degradation; and Grade 4: aortic rupture.

**Cell culture**

Different cell lines were chosen according to different purposes.

Primary Human Aortic Endothelial Cells (HAECs) was purchased from ScienCell Research Laboratories, which were cultured in Endothelial Cell Medium (ScienCell, 1001). To investigate the effect of Yoda-1 on endothelial barrier function, HAECs were stimulated with DMSO+Ang II (1 μM, MCE, HY-13948), Yoda-1 (10 μM, Selleck, s6678) +Ang II for 24 hours. To investigate the effect of Ang II on endothelial barrier function, HAECs were stimulated with saline+vehicle, saline+GsmTx4 (5 μM, Selleck, p1205), Ang II+vehicle, Ang II+GsmTx4. To explore whether STAT3 could regulate the expression of CCL2, transfected HAECs with STAT3 overexpression plasmid using Lipofectamine™ 2000 Transfection Reagent (Thermo Fisher Scientific, 11668019). Cells were then harvested for western blot or ELISA assay.

Human Monocyte Leukemia Cell line (THP-1) was purchased from iCell Cellverse (iCell-h213), which were cultured in RPMI-1640 Medium (iCell-0002). THP-1 cells were stimulated with PMA (200 ng/ml, Macklin, P849986) for 24 hours to induce macrophage-like monocytes, which were used for the subsequent endothelium-macrophage crosstalk experiments.

Mouse Monocyte/macrophage Leukemia Cells (RAW264.7) was purchased from iCell Cellverse (iCell-m047), which were cultured in Dulbecco's modified eagle medium (DMEM) supplemented with 10% fetal bovine serum. RAW Cells were co-cultured with ECs to investigate whether Piezo1 activation in ECs could regulate macrophages migration. Besides, to investigate whether macrophages could regulate CCR2 expression upon Piezo1 activation in ECs, RAW Cells were stimulated with Yoda-1-stimulated ECs supernatant and CCR2 inhibitor for 24 hours. Cells were then harvested for RT-qPCR.

**Extraction and cultivation human aortic endothelial cell**

The isolation of human aortic endothelial cells begins with pre-processing the obtained aortic segment, which is transported in cold antibiotic-containing DPBS, thoroughly rinsed, and carefully dissected to remove adventitial fat and connective tissue before being longitudinally opened to expose the intimal surface. For enzymatic digestion, the opened tissue is pinned intima-side up in a dish and covered with pre-warmed 0.1% collagenase solution, followed by incubation at 37°C for 30-60 minutes with periodic monitoring. Cells are then collected by gently scraping the digested intima and rinsing the tissue with FBS-containing solution to neutralize the enzyme and pool the suspensions. Finally, the cell suspension is filtered, centrifuged, and the pellet is resuspended in complete endothelial medium—with an optional red blood cell lysis step—before being seeded onto gelatin-coated vessels and cultured at 37°C/5% CO₂, with the medium replaced after 24 hours and subsequently every 2-3 days.

**Permeability assay**

HAEC monolayers were established on Transwell upper chamber. The test compounds were added to the upper chamber, and the cells were maintained under standard culture conditions.

After incubation, the monolayers were gently rinsed with PBS. Subsequently, 0.5 mL of fluorescein isothiocyanate (FITC) labeled dextran (0.5 g/L) was applied to the upper chamber, while 1.5 mL of dextran-free medium was added to the lower chamber. The system was protected from light and incubated for 1 hour.

Following incubation, 0.3 mL and 1.2 mL aliquots were collected from the upper and lower chambers, respectively. Fluorescence was measured using a microplate reader. A standard curve was generated using FITC-dextran standards, and the concentrations of FITC-dextran in both chambers were calculated accordingly.

**Adhesion experiment**

Monocyte-endothelial interactions were detected by [2',7'-bis (2-carboxyethyl)-5(6)-carboxyl fluorescein] acetoxymethyl ester (BCECF-AM, Sigma-Aldrich,14562)-labeled THP-1 cells adhering to HAECs. HAECs were cultured with or without Piezo1 inhibitor GsMTx4 for 30 minutes, and then incubated in serum-free medium containing Ang II for 24 hours. THP-1 cells were labeled with BCECF-AM for 30 minutes and co-cultured with the above HAEC for 5 hours. Clean non-adherent THP-1 cells. Adherent cells were counted in 5 random areas per well.

**Cell migration assay**

HAECs were inoculated into the lower chamber of transwell and cultured with corresponding stimulation. Macrophages were inoculated into the upper chamber of transwell for co-culture. The upper and lower cultures were separated by polycarbonate membranes. Due to the permeability of polycarbonate membranes, chemokines produced by endothelial cells in the lower medium could affect cells in the upper chamber. After 24 hours, the transwell chambers were removed, the culture medium in the hole was discarded, washed twice with PBS, and fixed with methanol for 30 minutes. After staining with 0.1% Giemsa dye solution at room temperature for 20 minutes, the upper layer of non-migrated cells was gently wiped off with a cotton swab and washed with PBS for 3 times. The cells were then observed in five fields under the microscope and counted.

**Construction of adeno-associated virus**

AAV serotype 1 (AAV1) vector serotypes (with an ICAM2 promoter) is the preferred viral vector to target aortic endothelial cell after injection. AAV1 shRNA STAT3 sequence was cloned into the pAV-ICAM2-GFP-mir30-shRNA to get STAT3-RNAi plasmid. AAV1 shRNA STAT3 sequence was cloned into the pAV-ICAM2-GFP-mir30-shRNA to get an STAT3-RNAi plasmid. Specific sequences and nonspecific controls were separately constructed in one vector, and AAV1 harboring these sequences were generated by Vigene Biosciences (Jinan, China). Mice were injected with one of the above viruses (5×10^11^ pfu/kg) for 3 weeks before they were randomly grouped and infused with Ang II. shRNA sequence is listed in Table S3.

**Real-time quantitative PCR (RT-qPCR)**

Total RNA was extracted from cells or tissues using Trizol Reagent (Sigma, T9424). Followed by reverse transcription into cDNA with the Reverse Transcription System kit (Vazyme, R223-01). The synthesized cDNA was amplified with a standard quantitative polymerase chain reaction (qPCR) protocol including the use of ChamQ Universal SYBR qPCR Master Mix (Vazyme, Q711-02). The amplified product was normalized with GAPDH mRNA to amplify in the same reaction as the internal control. The data were analyzed according to 2^−ΔΔCt^ method. The fold change in mRNA expression was quantified relative to control. RT-qPCR primers are listed in Table S3.

**Western blot**

Prepare the lysis buffer according to the ratio of RIPA Lysis Buffer (Beyotime, P0013B): Protease inhibitor (Boster, AR1182): Phosphatase inhibitor (Boster, AR1183) = 100:1:1. Tissue or cell lysates with the same protein content (determined by the BCA method; Boster, AR0146) were prepared. Proteins were separated by 10% SDS-PAGE or 12% SDS-PAGE and transferred to polyvinylidene difluoride (PVDF) membranes. After being blocked for 1.5 hours in 5% non-fat milk, the bands were incubated overnight at 4°C with primary antibodies. After washing, secondary antibodies were incubated for 1.5 hours. The bands were scanned and detected by a chemiluminescence instrument (General Electric Company, AI600RGB). The relative intensity of immunoreactive bands was assessed by Image J software. The results were normalized to GAPDH or β-Actin levels and expressed as % of control. All experiments were repeated at least thrice. Antibodies against Piezo1 (ab259949), VE-cadherin (ab33168), JAM-A (ab269948), claudin-5 (ab131259) were from Abcam (UK). Antibodies against IL-1β (#12242S), PYK2 (#3292), p-PYK2 (#3291), SRC (#2109), p-SRC (#6943), MLC (#3672), p-MLC (#3675), β-actin (#3700), STAT3 (#9139), p-STAT3 (#9145) were from Cell Signaling Technology (CST, Danvers, MA). The TNF-α antibody (sc-52746), p120-catenin antibody (sc-23873), AT1R antibody (sc-515884) were from Santa Cruz Biotechnology. p-VE-cadherin antibody (SAB4504676) was from Sigma Aldrich. Details of antibodies are listed in Table S4.

**The enzyme-linked immunosorbent assay (ELISA)**

After infusion, cell samples and cell culture mediums were collected and stored at −80 °C until further analysis. Following the manufacturers’ guidelines, the levels of CCL2 was quantified using ELISA kits.

**Immunofluorescence and confocal microscopy**

After being washed with PBS thrice, the cells coverslip or tissue sections were fixed with 4% paraformaldehyde in PBS for 20 minutes at room temperature. Aspirate fixative, rinse three times in PBS for 5 minutes each. Permeabilize samples with 0.1% Triton X-100 in PBS for 10 minutes. The cells coverslip or tissue sections were washed with PBS thrice, blocked with 5% bovine serum albumin for 30 minutes. Then incubated with the following primary antibodies diluted 1:100 at 4°C overnight: anti-Piezo1 (Abcam, ab259949), anti-CD31 (Thermo Fisher Scientific, MA1-26196), anti-CD68 (Abcam, ab955, ab53444), anti-α-SMA (Abcam, ab7817), anti-VE-cadherin (Abcam, ab33168), anti-p120-catenin (Santa Cruz Biotechnology, sc-23873), anti-MMP2 (Abcam, ab92536), anti-MCP-1 (Santa Cruz Biotechnology, sc-52701) and anti-TNF-α (Santa Cruz Biotechnology, sc-52746), anti-IL-1β (Cell Signaling Technology, #12242S). Subsequently, the cells coverslip or tissue sections were incubated with the appropriate secondary antibodies at room temperature for one hour. Finally, the cells coverslip or tissue sections were mounted with DAPI. Images of cells or tissue were taken under the confocal laser scanning fluorescence microscopy (LSM710; Carl Zeiss AG, Jena, Germany).

**Histological analysis**

The entire aorta was fixed with 10% formalin at room temperature for 24 hours, then paraffin sections were embedded. Tissues were sectioned at 5 μm and stained with H&E staining, Elastic tissue fibers-Verhoeff's Van Gieson (EVG) staining, Masson staining or immunohistochemical staining. After heat-mediated antigen retrieval, paraffin sections were blocked with 5% BSA and subjected to immunohistochemical staining. The sections were incubated overnight at 4°C with primary antibodies against Piezo1 (Abcam, ab259949). After several washes with PBS, the sections were incubated with biotin-labeled goat anti-rabbit and goat anti-mouse secondary antibodies at room temperature for one hour. Labeling was visualized with a 0.05% diaminobenzidine solution, and images of cells were taken under the confocal laser scanning fluorescence microscopy (Carl Zeiss AG, LSM710).

**Statistical analysis**

Continuous data were expressed as means±SEM and categorical data were expressed as numbers (%). The normality of data distribution was tested by using a Shapiro-Wilks test, then the Brown-Forsythe test was performed to check for equal variances among normally distributed data. Comparisons between groups were performed by Student t-test or one-way ANOVA followed by Tukey post hoc test if the assumption of equal variances is met. Non-parametric tests were used for data that were not normally distributed or the sample sizes were small. The Mann-Whitney test was applied for comparisons between the two groups. Kruskal-Wallis test with Dunn’s multiple comparisons test were applied for comparisons among more than two groups. The survival curve analysis was analyzed using the Kaplan-Meier product-limit approach and compared by the log-rank test. Statistical significance was considered at *p*<0.05. All data were analyzed using GraphPad Prism version 8.0. We downloaded publicly available data from the Gene Expression Synthesis (GEO) database (GSE232911 and GSE147026) and analyzed the correlation between Piezo1 expression with inflammation markers. The correlation between two genes was analyzed using the Pearson correlation.

**Data availability**

Data supporting the findings of this study are available within the article and its Supplementary Information files. Source data are provided with this paper.
